# Noncanonical Modulation of the eIF2 Pathway Controls an Increase in Local Translation during Neural Wiring

**DOI:** 10.1016/j.molcel.2018.11.013

**Published:** 2019-02-07

**Authors:** Roberta Cagnetta, Hovy Ho-Wai Wong, Christian K. Frese, Giovanna R. Mallucci, Jeroen Krijgsveld, Christine E. Holt

**Affiliations:** 1 Department of Physiology, Development and Neuroscience, Anatomy Building, University of Cambridge, Cambridge CB2 3DY, UK; 2 European Molecular Biology Laboratory (EMBL), 69117 Heidelberg, Germany; 3 German Cancer Research Center (DKFZ), 69121 Heidelberg, Germany; 4 CECAD Research Center, University of Cologne, 50931 Cologne, Germany; 5 UK Dementia Research Institute and Department of Clinical Neurosciences, Island Research Building, Cambridge Biomedical Campus, University of Cambridge, Cambridge CB2 0SL, UK

**Keywords:** unfolded protein response, Sema3A, PERK, eIF2α, eIF2B, local translation, axon, retinal ganglion cell, axon guidance, axon branching

## Abstract

Local translation is rapidly regulated by extrinsic signals during neural wiring, but its control mechanisms remain elusive. Here we show that the extracellular cue Sema3A induces an initial burst in local translation that precisely controls phosphorylation of the translation initiation factor eIF2α via the unfolded protein response (UPR) kinase PERK. Strikingly, in contrast to canonical UPR signaling, Sema3A-induced eIF2α phosphorylation bypasses global translational repression and underlies an increase in local translation through differential activity of eIF2B mediated by protein phosphatase 1. Ultrasensitive proteomics analysis of axons reveals 75 proteins translationally controlled via the Sema3A-p-eIF2α pathway. These include proteostasis- and actin cytoskeleton-related proteins but not canonical stress markers. Finally, we show that PERK signaling is needed for directional axon migration and visual pathway development *in vivo*. Thus, our findings reveal a noncanonical eIF2 signaling pathway that controls selective changes in axon translation and is required for neural wiring.

## Highlights

• eIF2α phosphorylation underlies Sema3A-induced upregulation of global translation
• pSILAC-SP3 reveals 75 nascent proteins regulated by the Sema3A-p-eIF2α pathway
• eIF2B modulation is the key node between Sema3A and canonical stress responses
• PERK signaling is required for axon guidance and neural connectivity

## Graphical Abstract

**Figure:**
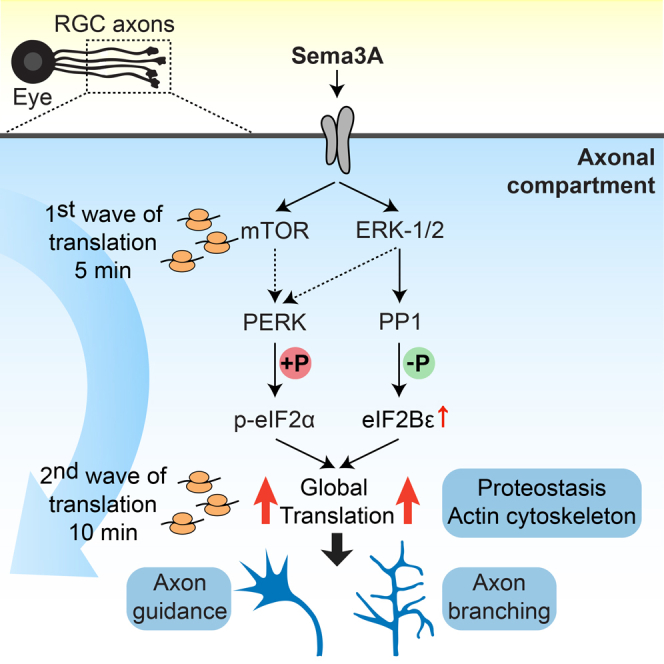

## Teaser

Cagnetta et al. find that Sema3A triggers phosphorylation of eIF2α in axons through an initial wave of local protein synthesis. eIF2α phosphorylation upregulates global translation via differential eIF2B activity and is required for neural wiring. Proteomics analysis identifies the axonal translational changes regulated by the Sema3A-p-eIF2α pathway.

## Introduction

Precise connectivity between neurons is needed to generate operative nervous systems. The initial assembly of neural circuits is mediated by the growth cone, a specialized structure at the tip of a growing axon that senses extracellular cues along the pathway and transduces them into directional changes, thus navigating to its synaptic partner (Stoeckli, 2018, Jung et al., 2012). When at the target, axons elaborate highly branched arbors and form synapses. The growth cone is often far from the soma, and local mRNA translation mediates its rapid responses to extracellular cues (Campbell and Holt, 2001, Jung et al., 2012). Extrinsic cues, such as Semaphorin-3A (Sema3A), specifically remodel the nascent axonal proteome within just 5 min and orchestrate further dynamic changes over the next 25 min (Cagnetta et al., 2018). However, the translational mechanisms that control the cue-induced local nascent proteome remain elusive.

One way to control translation is to modulate one or more components of the translational machinery. For instance, the α subunit of eukaryotic initiation factor 2 (eIF2α) mediates translational regulation in response to stress. eIF2α, guanosine triphosphate (GTP), and the methionyl initiator tRNA constitute the ternary complex (eIF2·GTP·tRNA_i_^Met^) that is delivered to the ribosome. As GTP is hydrolyzed during each round of translation initiation, eIF2 recharges via the guanine nucleotide exchange factor (GEF) eIF2B (Webb and Proud, 1997). Stress stimuli elicit phosphorylation of eIF2α at Ser51 via four possible kinases, including the PKR-like endoplasmic reticulum kinase (PERK) (Holcik and Sonenberg, 2005). Upon phosphorylation, p-eIF2α binds to and inhibits its own GEF, eIF2B, whose concentration is much lower than that of eIF2. Therefore, eIF2B can no longer return p-eIF2 to its active GTP-bound state (Webb and Proud, 1997). This causes a reduction of the ternary complex available to reinitiate translation, which represses the translation of most mRNAs and selectively upregulates a small subset of mRNAs (∼2.5% of total mRNAs; Dang Do et al., 2009). This mechanism allows the cell to conserve resources and to translate transcripts involved in the cytoprotective response or, when the stress is prolonged, in apoptosis (Holcik and Sonenberg, 2005). For instance, endoplasmic reticulum (ER) stress phosphorylates eIF2α via PERK to turn on the unfolded protein response (UPR), maintaining the homeostasis of the protein folding environment within the ER (Pavitt and Ron, 2012). Interestingly, Semaphorin signaling has been shown to govern epidermal morphogenesis via eIF2α dephosphorylation in *C. elegans* (Nukazuka et al., 2008), raising the possibility that Sema3A similarly employs the eIF2 pathway to control local translation-dependent axon guidance in vertebrate neurons.

Here we investigate the role of eIF2α in regulating the nascent proteome in the axonal compartment of retinal ganglion cells (RGCs) in response to Sema3A. Our findings reveal a noncanonical PERK-p-eIF2α signaling pathway that underlies the Sema3A-induced increase in local protein synthesis and is required for neural wiring. Further, our results identify eIF2B modulation as a pivotal switch between the responses to stress and Sema3A.

## Results

### Sema3A Induces eIF2α Phosphorylation in Axons

**Figure 1:**
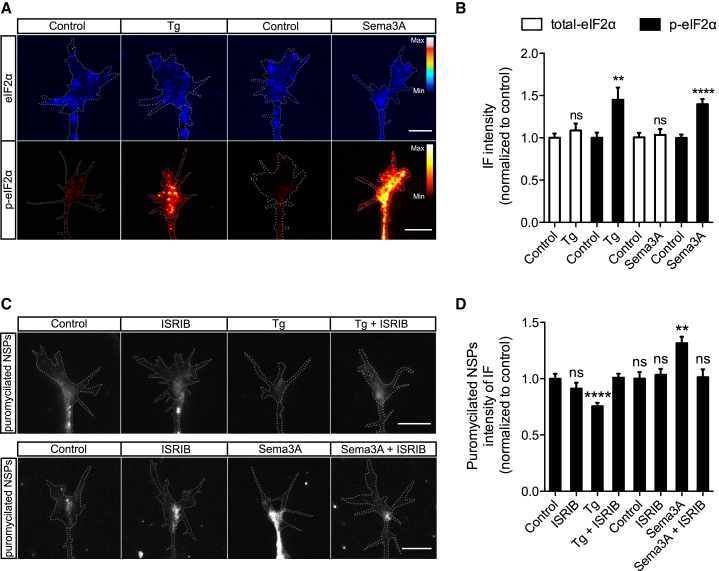
eIF2α Phosphorylation Underlies Sema3A-Induced Upregulation of Axonal Protein Synthesis (A and B) IF representative images (A) and quantification (B) for total-eIF2α and p-eIF2α in growth cones treated with Tg (15 min) or Sema3A (10 min) (unpaired t test). (C and D) IF representative images (C) and quantification (D) for puromycin in growth cones incubated with puromycin and co-treated with Tg (15 min) or Sema3A (10 min) and ISRIB (one-way ANOVA with Bonferroni’s multiple comparisons test). Error bars indicate SEM. Scale bars, 5 μm. See also Figure S1.

The extracellular cue Sema3A induces protein synthesis-dependent chemotropic responses in axonal growth cones, peaking 10 min after stimulation (Campbell and Holt, 2001, Campbell et al., 2001). Sema governs epidermal morphogenesis via eIF2α dephosphorylation in *C. elegans* (Nukazuka et al., 2008), prompting us to ask whether Sema3A similarly modulates eIF2α phosphorylation in axons. Quantitative immunofluorescence (qIF) revealed that Sema3A induces a significant increase in the p-eIF2α signal, but not in total-eIF2α, in retinal growth cones following 10 min stimulation (Figures 1A and 1B). The direction of the Sema-induced change in p-eIF2α was unexpectedly opposite to that seen in epidermal cells (Nukazuka et al., 2008) and was reminiscent of the p-eIF2α increase seen in the stress response. As a positive control, we compared the p-eIF2α signal in growth cones after stimulation with Sema3A versus treatment with the ER stress-inducing agent thapsigargin (Tg), an inhibitor of the sarco-endoplasmic reticulum Ca^2+^ ATPase (Vuppalanchi et al., 2012). Consistent with data from fibroblasts (Sadighi Akha et al., 2011), a 15 min treatment with Tg induced an increase in p-eIF2α, but not total-eIF2α, in axons (Figures 1A and 1B). Interestingly, in contrast to increased p-eIF2α levels that persist for hours in UPR signaling (Sadighi Akha et al., 2011), the increase with Sema3A treatment was rapid and transient, lasting minutes (Figure S1A). These data reveal that the physiological extracellular cue Sema3A triggers rapid and transient phosphorylation of eIF2α in axons.

### eIF2α Phosphorylation Differentially Regulates Translation in a Stimulus-Specific Manner

Sema3A increases global translation locally in retinal axons (Campbell and Holt, 2001, Yoon et al., 2012). However, paradoxically, Sema3A stimulation results in increased p-eIF2α, which is known to repress global translation (Holcik and Sonenberg, 2005). Therefore, we next explored the role of p-eIF2α on Sema3A-induced global translation in growth cones. To this end, newly synthesized proteins (NSPs) were tagged by puromycin pulse labeling (Schmidt et al., 2009). We stimulated with either Sema3A or the ER stressors Tg and DTT and co-treated with the pharmacological reagent integrated stress response inhibitor (ISRIB). ISRIB stabilizes eIF2B, making eIF2B’s GEF activity resistant to the effects of p-eIF2α without directly affecting eIF2α phosphorylation (Sidrauski et al., 2013, Sidrauski et al., 2015, Tsai et al., 2018). The released truncated puromycilated proteins were then quantified by IF using an anti-puromycin antibody. In accord with previous findings in whole cells (Sidrauski et al., 2013), Tg and DTT induced a decrease in the puromycin signal, signifying a decrease in global translation in the growth cone, which was blocked by ISRIB (Figures 1C, 1D, S1B, and S1C). Surprisingly, ISRIB completely abolished the Sema3A-induced increase in global translation, indicating that eIF2α phosphorylation also underlies the Sema3A-induced increase in protein synthesis in axons (Figures 1C and 1D). These results confirm that the stress response is conserved in axons (Vuppalanchi et al., 2012) and validate the mechanism of action of ISRIB in our system. Remarkably, they reveal that eIF2α phosphorylation can differentially regulate translation in a stimulus-specific manner.

### Sema3A Regulates a Specific Subset of Axonal NSPs via eIF2α Phosphorylation

**Figure 2:**
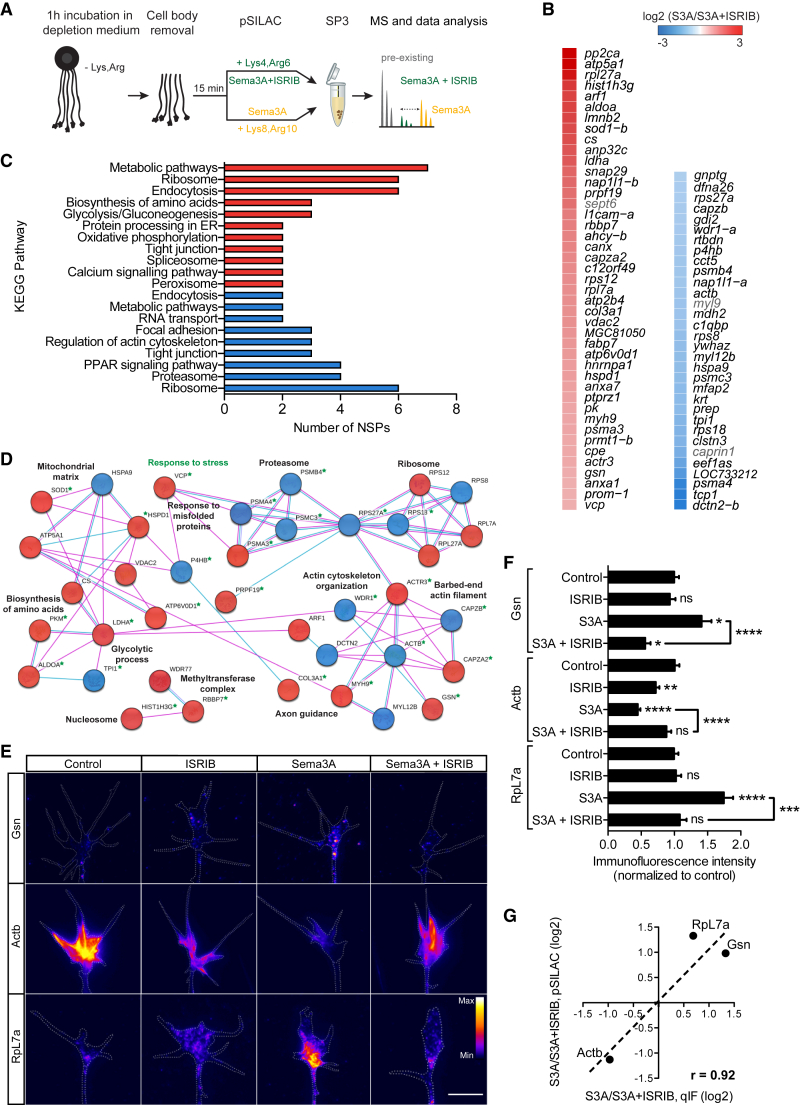
pSILAC-SP3 Reveals 75 Nascent Proteins Regulated by the Sema3A-p-eIF2α Pathway (A) Schematic of the pSILAC-SP3 methodology applied to somaless retinal axons. (B) Subset of NSPs regulated in response to Sema3A by p-eIF2α. Only significant NSP changes are shown (blue, downregulation; red, upregulation; p < 0.10). (C) KEGG pathway analysis (red, upregulated pathway; blue, downregulated pathway; cutoff ≥ 2 proteins per pathway). (D) Network-based cluster analysis of the enriched NSP changes induced by Sema3A-p-eIF2α signaling and their associated functional classes (blue nodes, downregulated NSPs; red nodes, upregulated NSPs; light blue edges, interactions known from databases; purple edges, interactions experimentally determined; green stars, NSPs belonging to the “response to stress” category; false discovery rate [FDR] < 0.05). (E and F) IF representative images (E) and quantification (F) for Gsn, Actb, and RpL7a in growth cones pre-incubated with ISRIB for 30 min and co-stimulated with Sema3A for 15 min (one-way ANOVA with Bonferroni’s multiple comparisons test). (G) Correlation analysis of pSILAC- and qIF-derived detection of protein changes (r = 0.92). Error bars indicate SEM. Scale bar, 5 μm. See also Figure S2 and Table S1.

We next investigated the proteins translationally regulated in the axon compartment of a single neuronal type (RGC) via the Sema3A-p-eIF2α pathway by employing pulsed stable isotope labelling of amino acids in cell culture together with single-pot solid phase-enhanced sample preparation (pSILAC-SP3; Hughes et al., 2014, Hughes et al., 2018, Cagnetta et al., 2018). RGC axons grown on transwell filters were incubated for 1 hr in depletion medium depleted of lysine and arginine and then severed from their cell bodies. Somaless axons were incubated for 15 min with Sema3A and “heavy” isotope-coded amino acids (Lys8 and Arg10) or with Sema3A, ISRIB, and “medium” isotope-coded amino acids (Lys4 and Arg6) (Figure 2A). ISRIB makes eIF2B insensitive to p-eIF2α, focusing the window of sensitivity of pSILAC-SP3 on the axonal NSPs regulated by the Sema3A-p-eIF2α pathway. pSILAC-SP3 revealed 75 significant NSP changes mediated by Sema3A-p-eIF2α signaling (Figure 2B; Table S1). Intriguingly, Atf4 mRNA is resident in axons (Zivraj et al., 2010), and the upstream open reading frames (uORFs) previously detected in the mouse are conserved in its 5′ UTR of *Xenopus laevis* and of mouse axons (Figures S2A and S2B). This leads to the prediction that Atf4 is upregulated when eIF2α is phosphorylated, and the level of ternary complex available to reinitiate translation decreases (Vattem and Wek, 2004). However, no upregulation of this classical stress marker was identified downstream of Sema3A-p-eIF2α signaling (Figure 2B; Table S1). This result was also confirmed by puromycilation of NSPs and proximity ligation assay (puro-PLA; tom Dieck et al., 2015) in the presence of Sema3A, whereas Tg treatment increased the Atf4 puro-PLA signal (Figures S2C and S2D), suggesting that the Sema3A-p-eIF2α pathway generates a level of ternary complex higher than the canonical stress response.

Kyoto Encyclopedia of Genes and Genomes (KEGG) pathway and functional gene ontology (GO) enrichment analyses revealed that the axonal NSPs regulated by Sema3A-p-eIF2α signaling encompass several functions (Figures 2C, 2D, and S2E). eIF2α phosphorylation upregulates the KEGG pathway “protein processing in ER,” including Canx and Vcp, which are involved in protein folding and quality control (Figure 2C). NSPs belonging to “metabolic pathways,” including proteins for the biosynthesis of amino acids, showed enhanced local translation (Figure 2C). Furthermore, eIF2α phosphorylation emerged to control the translation of some proteasomal subunits and ribosomal proteins (Figures 2C and S2E), possibly to remodel pre-existing proteasomes and ribosomes and confer substrate-specific functions (Shi et al., 2017, Padmanabhan et al., 2016). The Sema3A-p-eIF2α pathway also upregulates the “barbed-end actin filament” GO term (Figure S2E), including the actin-binding protein Gelsolin, which has been shown previously to mediate filopodium retraction (Lu et al., 1997). The translational upregulation of Gelsolin and of the ribosomal protein RpL7a concur with their mRNA presence in embryonic axons (Table S1; Gumy et al., 2011) and were validated by qIF (Figures 2E and 2F). Network-based functional enrichment analysis revealed that some NSP changes constitute functionally coherent sets of mRNAs undergoing differential translation regulation (Figure 2D). Interestingly, two upregulated NSPs, Hspd1 and Vcp, belong to the “response to misfolded proteins” GO term, and 31 NSPs are members of the “response to stress” GO term (Figure 2D). The Sema3A-p-eIF2α pathway also regulates several NSPs involved in “organization of the actin cytoskeleton,” including β-actin (Figure 2D), whose mRNA is present in retinal axons (Table S1; Zivraj et al., 2010) and whose downregulation was validated by qIF (Figures 2E and 2F). Collectively, comparison of the protein changes detected by pSILAC-SP3 versus the ones detected by qIF showed a strong positive correlation (r = 0.92; Figure 2G).

Next we tested whether the NSPs controlled downstream of the Sema3A-p-eIF2α pathway are predicted to be regulated by other *trans*-acting translational regulators. We performed an upstream regulator analysis based on previous datasets identifying targets of different translational regulators, including two canonical stress responses characterized in mouse liver downstream of the kinases PERK and GCN2. Only a very small fraction of targets was shared between Sema3A-p-eIF2α signaling and 4 of 8 translational regulators (PERK, GCN2, mTOR, and Apc) and more than 85% of the NSPs were predicted to be specifically regulated by the Sema3A-p-eIF2α pathway (Figure S2F). Alternatively, differences between experimental systems (e.g., transcriptome specificity) might contribute to this limited overlap. These results identify the subset of axonal NSPs selectively regulated by the Sema3A-p-eIF2α pathway.

### Sema3A-Induced Initial Wave of Local Translation Triggers eIF2α Phosphorylation via PERK

**Figure 3:**
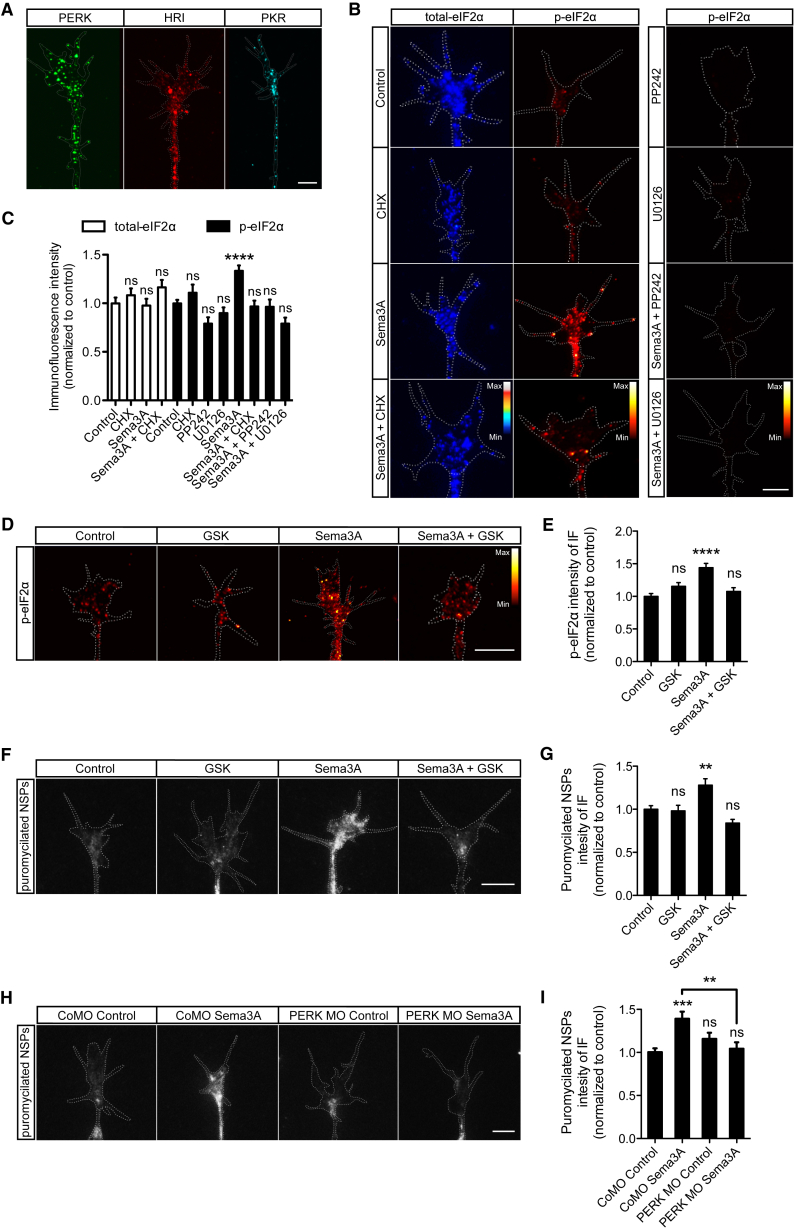
Sema3A-Induced Initial Wave of Local Protein Synthesis Elicits eIF2α Phosphorylation via PERK (A) Retinal axons were immunostained for PERK, HRI, and PKR. (B and C) IF representative images (B) and quantification (C) for total-eIF2α and p-eIF2α in growth cones co-treated with Sema3A and CHX, PP242, or U0126 for 10 min (one-way ANOVA with Bonferroni’s multiple comparisons test). (D and E) IF representative images (D) and quantification (E) for p-eIF2α in growth cones co-treated with Sema3A and GSK2606414 (GSK) for 10 min (one-way ANOVA with Bonferroni’s multiple comparisons test). (F and G) IF representative images (F) and quantification (G) for puromycin in growth cones co-incubated with puromycin, Sema3A and GSK for 10 min (one-way ANOVA with Bonferroni’s multiple comparisons test). (H and I) IF representative images (H) and quantification (I) for puromycin in growth cones of embryos injected with CoMO or PERK MO co-incubated with puromycin and Sema3A for 10 min (one-way ANOVA with Bonferroni’s multiple comparisons test). Error bars indicate SEM. Scale bars, 5 μm. See also Figure S3.

Four kinases are known to phosphorylate eIF2α (PERK, PKR, HRI, and GCN2) (Holcik and Sonenberg, 2005), prompting us to ask whether any of them are resident in retinal axons. PERK, PKR, and HRI are annotated in *X. laevis*, and IF indicated their presence in retinal axons (Figure 3A). Next, we asked which kinase is involved and how it is activated downstream of Sema3A. Within just 5 min (i.e., before eIF2α is phosphorylated; Figure S1A), Sema3A upregulates more than 60 significant axonal NSP changes without apparent links to proteostasis (Cagnetta et al., 2018). By contrast, at 15 min (i.e., after eIF2α is phosphorylated; Figure S1A), the Sema3A-p-eIF2α pathway upregulates NSPs linked to the biosynthesis of amino acids (e.g., Pkm), ER and mitochondrion protein quality control (e.g., the transitional ER ATPase Vcp), and chaperones (e.g., the ER chaperone Canx) (Figures 2B and 2D). This suggests a sequence of events in which an initial p-eIF2α-independent wave of translation places a burden on the ER, which, in turn, activates the kinase PERK to trigger a counteracting translational control program to preserve proteostasis. To test this hypothesis, we first blocked the Sema3A-induced burst in translation with the protein synthesis inhibitor cycloheximide (CHX) and immunostained for total-eIF2α and p-eIF2α. Remarkably, CHX completely blocked the Sema3A-induced phosphorylation of eIF2α without affecting the level of total-eIF2α (Figures 3B and 3C), indicating that upregulation of local translation is required for eIF2α phosphorylation. In particular, we tested whether the initial wave of local translation mediated by mTOR and ERK-1/2 (extracellular signal-regulated kinase, also known as mitogen-activated protein kinases p42 and p44), already active at 5 min of Sema3A stimulation (Campbell and Holt, 2001, Campbell and Holt, 2003), underlies the phosphorylation of eIF2α. Co-treatment of axons with Sema3A and the mTOR inhibitor PP242 or the ERK-1/2 inhibitor U0126 completely inhibited phosphorylation of eIF2α (Figures 3B and 3C), supporting the hypothesis. Each inhibitor on its own was sufficient to block eIF2α phosphorylation, suggesting that there is crosstalk between the ERK-1/2 and the mTOR pathways, in line with previous observations (Mendoza et al., 2011).

Finally, we stimulated axons with Sema3A in the presence of the PERK inhibitor GSK2606414 (GSK) and performed qIF for p-eIF2α. GSK blocked eIF2α phosphorylation (Figures 3D and 3E), revealing that PERK is activated by Sema3A. Consistent with our previous findings (Figures 1C and 1D), puromycilating the NSPs and co-treating with Sema3A and GSK phenocopied the effect of ISRIB by abolishing the Sema3A-induced increase in global translation (Figures 3F and 3G). To further verify these results, we knocked down PERK in embryos with a morpholino (MO), which resulted in ∼60% knockdown (KD) (Figure S3), and we assayed global translation in response to Sema3A stimulation. The Sema3A-induced increase in global translation was completely inhibited in PERK morphants (Figures 3H and 3I). Thus, PERK can be activated under physiological conditions following an initial wave of local translation and is a crucial component of the Sema3A pathway to upregulate local protein synthesis.

### Local Translation and Dephosphorylation of eIF2Bε Distinguish the Sema3A-Induced Response from the Canonical Stress Response

**Figure 4:**
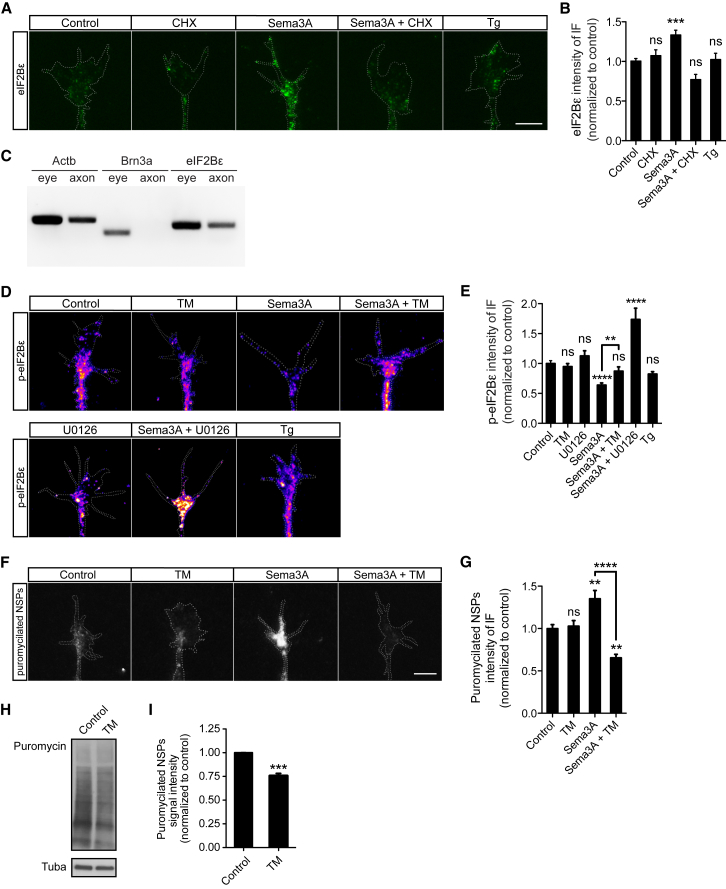
Sema3A and Canonical UPR Signaling Differentially Control Translation by Distinct Modulation of eIF2B (A and B) IF representative images (A) and quantification (B) for eIF2Bε in growth cones co-treated with Sema3A and CHX (10 min) or Tg (15 min) (one-way ANOVA with Bonferroni’s multiple comparisons test). (C) RT-PCR for Actb (positive control; Turner-Bridger et al., 2018), Brn3a (negative control; Yoon et al., 2012), and eIF2Bε mRNAs. (D and E) IF representative images (D) and quantification (E) for p-eIF2Bε (Ser539) in growth cones co-treated with Sema3A and tautomycin (TM) or U0126 (10 min) or treated with Tg (15 min) (one-way ANOVA with Bonferroni’s multiple comparisons test). (F and G) IF representative images (F) and quantification (G) for puromycin in growth cones co-incubated with puromycin, Sema3A and TM for 10 min (one-way ANOVA with Bonferroni’s multiple comparisons test). (H and I) Immunoblot (H) and quantification (I) of puromycin signal intensity in lysates of intact brains of stage 35-36 embryos incubated with TM for 30 min and puromycilated over the last 15 min of the treatment (unpaired t test). Error bars indicate SEM. Scale bars, 5 μm. See also Figure S4.

Our findings revealed that the Sema3A response and the canonical UPR are both mediated by phosphorylation of eIF2α. Therefore, we investigated the mechanism underlying the differential translational control downstream of these two stimuli. p-eIF2α induced by Sema3A signaling, unlike the canonical UPR, does not induce global translational repression (Figures 1C and 1D) nor the translation of classical stress markers such as Atf4 (Figures 2B and S2A–S2D), both of which are triggered by low levels of ternary complex. eIF2 recharges with GTP by eIF2B, which constitutes a rate-limiting factor for ternary complex availability (Webb and Proud, 1997). Therefore, we hypothesized that, while inducing phosphorylation of eIF2α, Sema3A may concomitantly modulate the GEF activity of eIF2B to alter the probability of generating the ternary complex. We reasoned that the modulation of eIF2B activity could be achieved in two non-mutually exclusive ways: by increasing the total amount of eIF2B available and by adjusting the phosphorylation level of a conserved Ser residue on the subunit ε of eIF2B, wherein phosphorylation decreases eIF2B activity, whereas dephosphorylation increases eIF2B activity (Welsh et al., 1998, Quevedo et al., 2003). We first examined the level of total eIF2Bε in the growth cone following Sema3A versus Tg treatment. qIF showed a more than 35% increase in total eIF2Bε in response to 10 min Sema3A but not Tg (Figures 4A and 4B). This rapid increase could result from local translation because eIF2Bε mRNA resides in retinal axons (Figure 4C). CHX blocked the Sema3A-induced increase in eIF2Bε (Figures 4A and 4B), indicating that eIF2Bε, unlike eIF2α (Figures 3B and 3C), is locally translated in response to Sema3A. This finding is in accord with the detection of eIF2Bε mRNA translation in mouse retinal axons in the tectum *in vivo*, where Sema3A is expressed (Shigeoka et al., 2016). Like reticulocytes, where the eIF2:eIF2B ratio is 7:1 (Webb and Proud, 1997), eIF2Bε is much less abundant than eIF2α in axons, as indicated by the inability of the pSILAC-SP3 approach to detect either nascent or pre-existing eIF2Bε protein, whereas eIF2α protein is readily detected (Cagnetta et al., 2018). Because Sema3A is known to activate axonal mTOR and ERK-1/2 within 5 min stimulation (Campbell and Holt, 2001, Campbell and Holt, 2003), we asked whether these translational regulators control eIF2Bε rapid local translation. The results showed that eIF2Bε increases within just 5 min of Sema3A stimulation, but this is not mediated by mTOR or ERK-1/2 (Figures S4A and S4B). The negative control showed that co-treatment with ISRIB does not affect the eIF2Bε increase (Figures S4A and S4B), consistent with the lack of eIF2α phosphorylation at 5 min stimulation (Figure S1A). Upstream regulator analysis based on previous datasets identifying the targets of several translational regulators predicted that Apc, Mena, Fmrp, Tdp43, Fus, or mTOR do not control eIF2Bε translation.

We next examined the level of phosphorylation of eIF2Bε, which regulates eIF2B activity. We stimulated with Sema3A for 10 min and immunostained growth cones for p-eIF2Bε (Ser539). qIF showed an ∼35% decrease in p-eIF2Bε in response to Sema3A but not Tg (Figures 4D and 4E). Previous work in rat cortical neurons has shown that dephosphorylation of p-eIF2Bε can be mediated by protein phosphatase 1 (PP1) activation following its interaction with ERK-1/2 (Quevedo et al., 2003), which constitutes a major component of the Sema3A intracellular pathway (Campbell and Holt, 2003). Therefore, we tested whether PP1 is responsible for eIF2Bε dephosphorylation by co-treating with Sema3A and tautomycin (TM), which preferentially inhibits PP1 (MacKintosh and MacKintosh, 1994). TM abolished the Sema3A-induced dephosphorylation of eIF2Bε (Figures 4D and 4E), revealing that Sema3A activates PP1 to mediate eIF2Bε dephosphorylation. In contrast, PP1 does not regulate the eIF2α phosphorylation level (Figures S4C and S4D), indicating a lack of involvement of the PP1-eIF2α-recruiting scaffold protein GADD34 (growth arrest and DNA damage-inducible protein) at 10 min Sema3A stimulation (Choy et al., 2015). Finally, we tested whether ERK-1/2 is upstream of eIF2Bε dephosphorylation by co-treating with Sema3A and U0126 and immunostaining for p-eIF2Bε. Interestingly, the results revealed that the dephosphorylation of eIF2Bε switches to phosphorylation (Figures 4D and 4E). This, together with previous studies in cortical neurons, suggests that ERK-1/2 activates PP1 and simultaneously suppresses the activity of GSK-3β (Quevedo et al., 2003, Hetman et al., 2002), which is known to phosphorylate eIF2Bε and to be repressed at low Sema3A concentrations in a dose-dependent manner (Welsh et al., 1998, Manns et al., 2012).

To further test whether eIF2B activity represents a key node between the UPR and Sema3A differential translation control, we asked whether it is possible to switch the Sema3A-induced global translation upregulation to repression by only modulating eIF2B activity. To this end, we puromycilated the NSPs and stimulated with Sema3A in the presence of TM, which inhibits eIF2Bε dephosphorylation (Figures 4D and 4E) and blocks its increase in activity. Strikingly, the Sema3A-induced rapid increase in global translation switched to repression (Figures 4F and 4G), mimicking the effects of the Tg- and DTT-induced stress response (Figures 1C and 1D; Figures S1B and S1C). This result indicates that the Sema3A-induced local increase in total-eIF2Bε (Figures 4A and 4B) on its own is not sufficient to increase eIF2B overall GEF activity and that Sema3A-induced phosphorylation of eIF2α underlies the increase in global translation by engaging eIF2Bε dephosphorylation. Finally, we asked whether this translational control mechanism is also detectable in the developing nervous system *in vivo*. We incubated brains of embryos at a stage when Sema3A is known to act on retinal axons (stage 35/36; Campbell et al., 2001) with TM for 30 min, puromycilated NSPs over the last 15 min of the treatment, and carried out western blotting to probe for puromycin. The results showed a decrease in global translation equal to ∼25% (Figures 4H and 4I), indicating that translation mediated by p-eIF2α-eIF2Bε signaling occurs in the developing brain *in vivo*. Altogether, the findings indicate that, during neurodevelopment, Sema3A-induced phosphorylation of axonal eIF2α underlies the noncanonical increase in global translation by enhancing eIF2B activity primarily through dephosphorylation of its ε subunit.

### Sema3A-Induced Polarized Phosphorylation of eIF2α Is Required for Directional Migration

**Figure 5:**
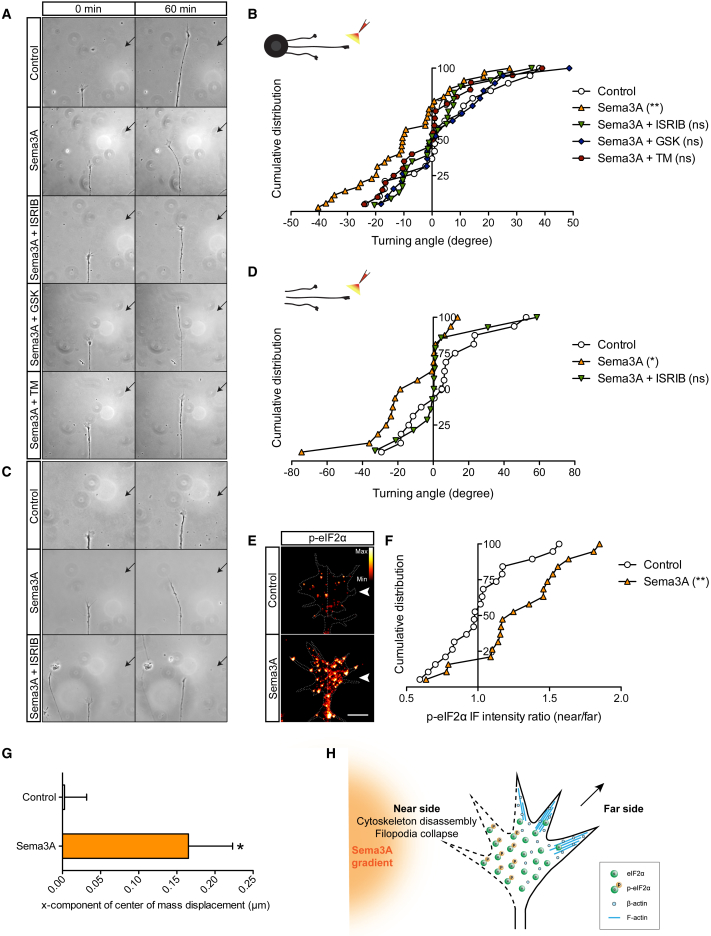
Spatially Polarized Phosphorylation of eIF2α Mediates Sema3A-Induced Chemorepulsion (A) Turning assay. Arrows indicate the position of the pipette. (B) Cumulative distribution of turning assay outcome. A polarized gradient of Sema3A was generated, and ISRIB, GSK, or TM were bath-applied. Positive values indicate attraction, and negative values indicate repulsion (unpaired t test). (C) Turning assay with somaless axons. Arrows indicate the position of the pipette. Eye explants were removed immediately prior to the experiment. (D) Cumulative distribution of turning assay outcome. A polarized gradient of Sema3A was generated, and ISRIB was bath-applied. Positive values indicate attraction, and negative values indicate repulsion (unpaired t test). (E) Growth cone immunostained for p-eIF2α with a line dividing the near and far sides. Arrowheads indicate the 90° polarized gradient of Sema3A. (F) Cumulative distribution assessing the asymmetric increase of p-eIF2α with the near:far ratio method (unpaired t test). (G) Asymmetric increase of p-eIF2α, assessed by the center of mass method (unpaired t test). (H) Sema3A-induced repulsive growth cone model. p-eIF2α increases on the near-stimulus side, controlling the β-actin polarized decrease (Cagnetta et al., 2018), thus helping with asymmetric cytoskeleton deconstruction and filopodium collapse. Error bars indicate SEM. Scale bar, 5 μm. See also Figure S5.

We next explored the functional significance of eIF2α phosphorylation downstream of Sema3A. pSILAC-SP3 had revealed that Sema3A-p-eIF2α signaling controls the translation of several proteins involved in the regulation of the actin cytoskeleton and in axon guidance (Figure 2D). For example, the Sema3A-p-eIF2α pathway upregulates Gelsolin (Figures 2B, 2E, and 2F), an actin-binding protein required for filopodium retraction (Lu et al., 1997), and L1cam (Figure 2B), a cell adhesion molecule necessary for topographic mapping of retinal axons (Demyanenko and Maness, 2003). Therefore, we investigated whether phosphorylation of eIF2α plays a role in Sema3A-induced chemorepulsion. We carried out growth cone turning assays with a polarized gradient of Sema3A and bath-applied ISRIB or GSK. Both treatments blocked Sema3A-induced repulsive turning (Figures 5A, 5B, and S5A). In somaless axons, repulsive turning was also inhibited by ISRIB (Figures 5C, 5D, and S5B), indicating that local phosphorylation of eIF2α mediates Sema3A-induced chemorepulsion. Because the Sema3A-p-eIF2α-mediated increase in translation is dictated by eIF2Bε dephosphorylation (Figures 4D and 4G), we tested whether blocking PP1 also affects Sema3A-induced repulsive turning. Bath application of TM inhibited chemorepulsion (Figures 5A, 5B, and S5A).

In light of these results, we reasoned that, during the chemotropic response, a directional stimulus of Sema3A might produce a polarized asymmetrical phosphorylation of eIF2α, generating an internal gradient of proteomic change across the growth cone. To test this possibility, a gradient of Sema3A was applied for 10 min at a 90° angle to the growth cone as an assay to achieve a steep difference between the “near” and the “far” sides of the growth cone (Figure 5E). qIF indicated that a Sema3A gradient significantly increases eIF2α phosphorylation on the near-stimulus side (Figures 5E, 5F, and S5C). Further confirmation of the asymmetrical phosphorylation was obtained by comparing the center-of-mass value of the p-eIF2α IF signal between the control and Sema3A gradient conditions, which revealed an equivalent shift toward the Sema3A source (Figures 5G and S5D). These results, together with the finding that Sema3A downregulates β-actin via p-eIF2α (Figures 2B, 2E, and 2F), are consistent with previous work showing that β-actin decreases on the near-stimulus side in response to a polarized gradient of Sema3A (Cagnetta et al., 2018) and support a growth cone-repulsive model where p-eIF2α increases on the side close to the source of Sema3A to mediate cytoskeleton disassembly and filopodium collapse (Figure 5H). Collectively, the data show that polarized phosphorylation of eIF2α within the growth cone is required for Sema3A-induced directional migration.

### PERK Signaling Is Involved in Retinotectal Axon Navigation *In Vivo*

**Figure 6:**
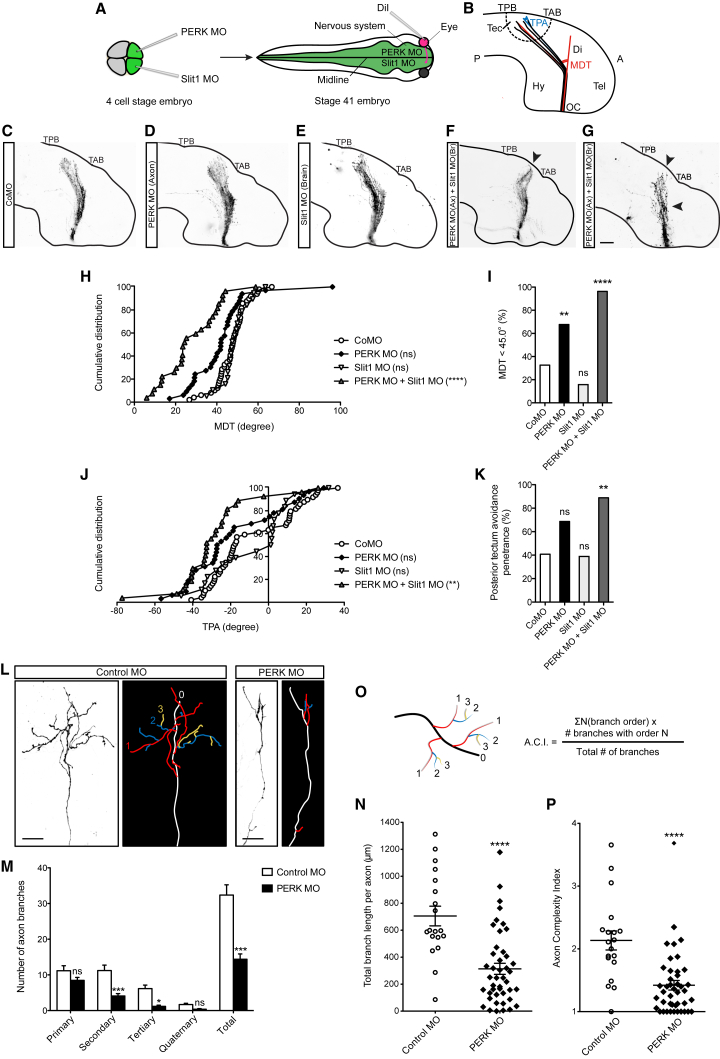
PERK Signaling Is Required for Visual Pathway Development *In Vivo* (A) Experimental outline to investigate the contribution of axonal PERK in RGCs only and Slit1 in the optic tract pathway substrate. Unilateral MO injection leads to targeted KD in half of the nervous system. (B) Schematic of axons navigating the optic tract and reaching the tectum. TPB, tectal posterior boundary; TAB, tectal anterior boundary; TPA tectal projection angle; MDT, mid-diencephalic turn; A, anterior; P, posterior; OC, optic chiasm; Tec, tectum; Di, diencephalon; Hy, hypothalamus; Tel, telencephalon. (C–G) Representative images of DiI-filled stage 41 retinotectal projections in Control MO (C), unilateral KD of PERK in the axons (D), unilateral KD of Slit1 in the optic tract substrate (E), or both (F and G) (Ax, axon; Br, brain). (H) Cumulative distribution of MDT angle measurements in unilateral KD of PERK in the axons or Slit1 in the optic tract substrate or both (one-way ANOVA with Bonferroni’s multiple comparisons test). (I) Penetrance for MDT angles of less than 45° (Fisher’s exact test). (J) Cumulative distribution of TPA measurements in unilateral KD of PERK in the axons or Slit1 in the optic tract substrate or both. Positive values indicate angles pointing toward the TPB, and negative values indicate angles pointing toward the TAB (one-way ANOVA with Bonferroni’s multiple comparisons test). (K) Penetrance of posterior tectum avoidance, measured as TPA < mean TPA in CoMO (i.e., −8.6°) (Fisher’s exact test). (L) Single RGC axons in the tectum and line drawings of the corresponding trajectories shown by color-coded branch order: white, axon shaft; branches: red, primary; blue, secondary; yellow, tertiary. (M) Number of axon branches in the various orders and total number of branches in the PERK morphants (two-way ANOVA). (N) Length of axon branches in the PERK morphants (unpaired t test). (O) Formulation of axon complexity index (ACI). Color indicates the branch order as in (L). (P) ACI in the PERK morphants (Fisher’s exact test). Error bars indicate SEM. Scale bars, 100 μm (C–G) and 20 μm (L). See also Figure S6.

We next investigated whether eIF2α phosphorylation is involved in the navigation of retinal axons *in vivo*. The PERK MO was injected into only one of the two dorsal blastomeres, leading to embryos in which one half of the CNS is depleted of PERK and the other half is wild-type (Figure 6A; Roque et al., 2016). During development, RGC axons cross the midline at the optic chiasm and project contralaterally; therefore, 1,1′-Dioctadecyl-3,3,3′,3′-tetramethylindocarbocyanine perchlorate (DiI) injection in the ipsilateral eye enabled us to test the contribution of axonal PERK in RGCs only without affecting the optic tract substrate (Figures 6A and 6B). Embryos injected with PERK MO overall appeared to develop normal projections as in the control MO (CoMO) (Figures 6C and 6D), showing no difference in optic tract width (Figures S6A and S6B). PERK morphants exhibited a slight decrease (not statistically significant) in the mid-diencephalic turn (MDT) angle (Figures 6B, 6H, and S6C) and an increase in the proportion of embryos with an MDT angle smaller than that in the wild-type (i.e., MDT < 45°) (Figure 6I). Measurement of the tectal projection angle (TPA) (Figure 6B) revealed an increased tendency of axons to turn away from the posterior tectal border (not statistically significant; Figures 6J, 6K, and S6D). Collectively, this result is in line with previous studies showing, in the same *Xenopus* visual system, that no gross abnormalities were observed in axon navigation after either Sema3A KD or acute inhibition of protein synthesis (Atkinson-Leadbeater et al., 2010, Wong et al., 2017). Similarly, the retinotectal projection did not exhibit evident defects after genetic deletion or pharmacological inhibition of mTOR in zebrafish (Cioni et al., 2018).

Previous work has shown that Sema3A and Slit1 transcripts are both present at the mid-diencephalic turn and in the tectum and that these two cues cooperate to guide the turning of axons caudally (Campbell et al., 2001, Hocking et al., 2010, Atkinson-Leadbeater et al., 2010). Furthermore, a recent study of mouse dorsal root ganglion growth cones found that Sema3A and Slit1 induce chemorepulsion through distinct mechanisms (McConnell et al., 2016). Therefore, we reasoned that Slit1 might act via a PERK-independent route and compensate PERK KD downstream of Sema3A. We first tested whether Slit1 affects eIF2α phosphorylation. Interestingly, qIF showed no change in growth cone basal p-eIF2α level after Slit1 stimulation (Figures S6E and S6F), indicating that PERK is selectively activated downstream of Sema3A. Next, we tested Slit1 MO in our system (∼55% KD) (Figure S6G) and knocked down Slit1 in the optic tract substrate *in vivo* (Figure 6A). Consistent with previous results (Atkinson-Leadbeater et al., 2010), Slit1 KD did not interfere with optic tract width (Figures 6C, 6E, and S5B) or with navigation (Figures 6C, 6E, 6H–6K, S6C, and S6D). We then simultaneously knocked down PERK in the axon and Slit1 in the optic tract substrate (Figure 6A). Remarkably, DiI axon labeling revealed that, although the optic tract width remained unaffected (Figure S6B), the whole axonal bundle failed to turn caudally (Figures 6F–6I and S6C) and did not correctly enter the tectum in the midbrain (Figures 6F, 6G, 6J, 6K, and S6D).

Further, we exposed the intact brain to ISRIB treatment by removal of the overlying epidermis during the period of optic pathway formation (Figure S6H). In line with the PERK morphants, the results showed no significant difference in optic tract width, MDT angle, and tectal entry (Figures S6I, S6J, S6L, and S6M). When ISRIB treatment was combined with Slit1 KD in the optic tract substrate (Figure S6H), the brains exhibited axon guidance defects that phenocopied those seen with PERK-Slit1 KD (Figure S6K, S6M, and S6N) without affecting optic tract width (Figure S6L). Collectively, the data indicate that PERK-p-eIF2α signaling cooperates with other p-eIF2α-independent pathways in guidance cue integration during retinotectal axon navigation *in vivo*.

### PERK Signaling Is Required for Axon Terminal Branching *In Vivo*

Upon reaching the tectum, where Sema3A is expressed (Campbell et al., 2001), RGC axons elaborate terminal branches and form synapses. Sema3A has been shown to elicit branching of retinal and GABAergic axons (Campbell et al., 2001, Cioni et al., 2013), and axon branching is dependent on local protein synthesis *in vivo* (Wong et al., 2017), leading us to ask whether eIF2α phosphorylation is required for branching *in vivo*. The PERK MO and a membrane-targeted GFP (mGFP) reporter were co-electroporated into the eye at stage 28, and single axon arbors were imaged at stage 45 (Wong et al., 2017). Although CoMO-electroporated axons exhibited complex arbors, PERK MO axons exhibited a much simpler arbor architecture (Figure 6L). Quantitative analysis revealed that the branch numbers decreased across different branch orders, leading to an overall drop of 56% (Figure 6M). Furthermore, a 55% reduction of the total branch length was observed (Figure 6N). The axon complexity index (ACI) (Figure 6O; Marshak et al., 2007) showed a marked decrease in the PERK morphants (Figure 6P). These data reveal that PERK signaling is required for developing axon arbor complexity *in vivo*.

## Discussion

Extracellular stimuli can rapidly remodel the local nascent proteome in axons, and here we investigated the underlying translational control mechanisms and mRNA targets. We used nascent proteome analysis combined with *in vitro* and *in vivo* models to demonstrate that a Sema3A-induced initial wave of local translation triggers a noncanonical eIF2 signaling pathway. Subsequently, this pathway upregulates local translation and orchestrates a set of proteomic changes required for axon guidance and neural connectivity.

**Figure 7:**
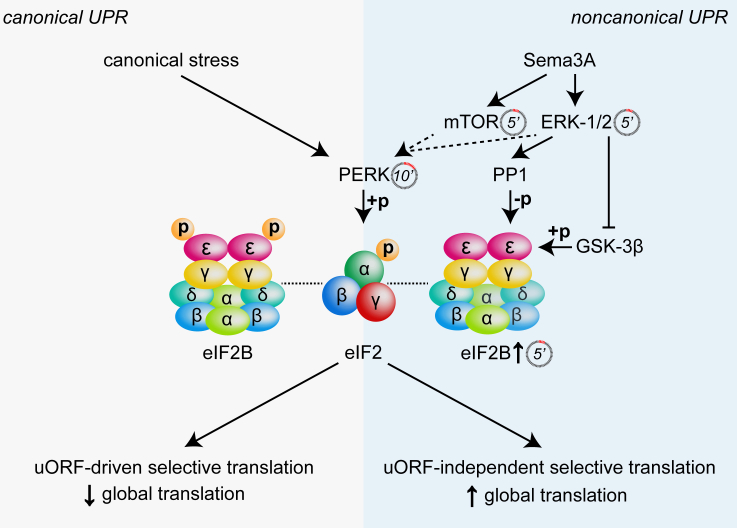
eIF2Bε Constitutes a Pivotal Node between the Responses to Canonical Stress and Sema3A Sema3A induces an initial (5 min) wave of local translation independent of the eIF2 pathway mediated by ERK-1/2 and mTOR. Simultaneously, eIF2Bε is locally translated in an ERK-1/2-mTOR-independent manner. The rapid increase in local protein synthesis triggers eIF2α phosphorylation via PERK at 10 min stimulation. Within this time course, ERK-1/2 represses GSK-3β and activates PP1, dephosphorylating eIF2Bε and increasing eIF2B activity. The engagement of p-eIF2α and increased eIF2B GEF activity generates a specific level of ternary complex higher than in the canonical stress response, resulting in uORF-independent selective translation of 75 NSPs, upregulating global translation. +p, phosphorylation, −p, dephosphorylation, ↑ and **↓**, axonal translation upregulation and downregulation; dashed lines, indirect activation following a rise in local protein synthesis; dotted lines, interaction; dashed circles, timing.

A canonical role for eIF2α phosphorylation under physiological conditions, rather than in response to stress or in pathology, has already emerged from recent studies (Di Prisco et al., 2014, Dalton et al., 2013, Trinh et al., 2014, Woo et al., 2012). Our work differs by showing, for the first time, that eIF2α phosphorylation can underlie an increase in translation as opposed to the decrease characterizing the canonical stress model. Notably, our results show that eIF2α phosphorylation (10 min) is dependent on the Sema3A-induced initial wave (≥5 min) of protein synthesis mediated by mTOR and ERK-1/2 (Figure 7). These findings support a model where the ER, known to reside throughout the axon (Luarte et al., 2018), becomes overloaded with new unfolded (i.e., yet to be folded) proteins following Sema3A stimulation (∼30% increase in global translation in only 10 min). This may cause physiological stress, which activates the ER stress sensor PERK and leads to eIF2α phosphorylation. This model is further supported by the downstream selective translation of NSPs involved in protein folding, ER and mitochondria protein quality control, and biosynthesis of amino acids, possibly to sustain the burst in global translation. Interestingly, ERK-1/2 also controls eIF2Bε, likely by activating PP1 and suppressing GSK-3β (Figure 7; Quevedo et al., 2003, Hetman et al., 2002). Therefore, the findings reveal a dependency between the pathways triggered downstream of Sema3A, wherein p-eIF2α-eIF2Bε signaling can be activated at the second stage of a cascade, and account for the dynamic and changing nature of the nascent axonal proteome during the 30 min post-stimulation (Cagnetta et al., 2018).

The phosphorylation status of eIF2Bε, and, hence, the GEF activity of eIF2B (Quevedo et al., 2003), dictates the outcome of the global translation levels distinguishing the response to Sema3A from the canonical UPR. Specifically, we found that Sema3A, unlike Tg, induces local translation and dephosphorylation of eIF2Bε via PP1 (Figure 7). The absence of Atf4 upregulation suggests that Sema3A-induced regulation of eIF2B and eIF2α phosphorylation precisely influences the rate of generation of the ternary complex, promoting higher exchange of guanosine diphosphate (GDP) for GTP on eIF2 than in the canonical stress response, which is instead triggered by low levels of ternary complex (Vattem and Wek, 2004). One possibility is that increased eIF2B activity exchanges GDP for GTP with higher efficiency than in the canonical UPR on the subpopulation of eIF2 that escapes phosphorylation on its α subunit. A further possibility is that dephosphorylation of eIF2Bε may stabilize the eIF2B dimer, decreasing its affinity for p-eIF2α and permitting higher GDP-GTP exchange than in the canonical UPR, similar to the mechanism of action of ISRIB (Tsai et al., 2018, Sidrauski et al., 2015). This bypasses the global translational repression and the uORF-mediated upregulation of canonical stress markers and regulates the translational efficiency of a subset of mRNAs possibly sensitive to such levels of ternary complex (Figure 7). It is interesting to speculate that this translational control mechanism may also be employed by other biological processes to tackle large increases in protein synthesis (Baleriola et al., 2014) and consequent ER overload, bypassing the translation of pro-apoptotic factors (e.g., Chop; Woo et al., 2012) and regulating the translation of a specific subset of mRNAs (e.g., for proteostasis). Furthermore, this noncanonical way to control translation may suggest new therapeutic targets for disorders involving the detrimental expression of UPR markers and pathogenic translation repression (Moreno et al., 2012, Ma et al., 2013).

Our study also revealed that a physiological extracellular stimulus can control the phosphorylation of eIF2α in axons with spatio-temporal precision. The phosphorylation is polarized to the near-stimulus compartment of the growth cone, indicating that its translational control mechanism can be further spatially compartmentalized at the subcellular level (Figure 5H). Of particular interest is that the Sema3A-induced phosphorylation of eIF2α is transient, peaking 10 min post-stimulation and declining thereafter, which is in contrast to the canonical stress response, where eIF2α phosphorylation typically peaks at 30 min and endures for hours (Sadighi Akha et al., 2011). The phosphatase PP2Cα topped the list of selectively upregulated nascent proteins in response to Sema3A via p-eIF2α. PP2Cα dephosphorylates and, thereby, inhibits the 5′ adenosine monophosphate-activated protein kinase (AMPK) (Lammers and Lavi, 2007), which has been reported to be required for PERK activation in a specific UPR pathway but not in response to Tg (Yang et al., 2013). This raises the possibility that Sema3A turns off the PERK-p-eIF2α pathway with a built-in negative feedback loop by triggering the local translation of PP2Cα, thus accounting for the eIF2α transient phosphorylation. This fast mechanism could accommodate the rapid cue-induced response locally, likely required *in vivo* for the growing axons to make timely navigational and connectivity decisions.

PERK signaling is involved in axon retinotectal navigation *in vivo* by working at the mid-diencephalic turn and in the tectum together with Slit1, whose downstream signaling pathway is p-eIF2α-independent. This mechanism may have evolved to build a more robust system and increase axon navigation accuracy. PERK signaling is also required for axon arbor formation in the tectum, in line with recent *in vivo* evidence showing that acute inhibition of protein synthesis impairs axon terminal branching (Wong et al., 2017). Importantly, given that the axons are exposed to various guidance cues in the tectum, we do not exclude the interesting possibility that PERK may act downstream of further extracellular stimuli that, like Sema3A, induce a strong global increase in local translation (Yoon et al., 2012).

Last, some of the NSPs regulated via Sema3A-p-eIF2α signaling are neurological disease-associated (Table S2), suggesting links between defective axonal translational control in neural wiring and disease. In addition, Sema3A is known to inhibit axon regeneration following injury in the adult nervous system (Giger et al., 2010); hence, the eIF2 pathway may represent a therapeutic target for neural repair. In conclusion, the noncanonical signaling reported could open new avenues of investigation in translational control and lead to a better understanding of neural wiring and potentially help with the development of new therapeutic approaches.

## STAR★Methods

### Key Resources Table

**Table:** 

REAGENT or RESOURCE	SOURCE	IDENTIFIER
**Antibodies**		

anti-eIF2α	Abcam	Cat#ab137626; RRID: AB_2736873
anti-p-eIF2α	Abcam	Cat#ab32157; RRID: AB_732117
anti-puromycin Alexa Fluor 488 conjugate	Millipore	Cat#MABE343-AF488; RRID: AB_2736875
anti-puromycin Alexa Fluor 647 conjugate	Millipore	Cat#MABE343-AF647; RRID:AB_2736876
anti-puromycin	Millipore	Cat#MABE343; RRID: AB_2566826
anti-Actb	Abcam	Cat#ab6277; RRID: AB_305394
anti-Gsn	Proteintech	Cat#11644-2-AP; RRID: AB_2295090
anti-RpL7a	Abcam	Cat#ab155147; RRID: AB_2736874
anti-Atf4	Abcam	Cat# ab85049; RRID: AB_1861369
anti-eIF2Bε	Abcam	Cat#ab32713; RRID: AB_2230901
anti-p-eIF2Bε (Ser539)	Proteintech	N/A (customized)
anti-PERK	Cell signaling	Cat#3192; RRID: AB_2095847
anti-Tuba	Sigma	Cat#T6074; RRID: AB_477582
anti-Slit1	Abcam	Cat# ab115892; RRID: AB_10903854

**Chemicals, Peptides, and Recombinant Proteins**		

Sema3A	R&D Systems	Cat#1250-S3
Slit1	R&D Systems	Cat#6514-SL
ISRIB	Sigma	Cat#SML0843
Thapsigargin	Sigma	Cat#T9033
Dithiothreitol	Sigma	Cat#D0632
GSK2606414	Calbiochem	Cat#516535
Cycloheximide	Sigma	Cat#C4859
PP242	Tocris	Cat#4257
U0126	Tocris	Cat#1144
Tautomycin	Calbiochem	Cat#580551
Poly-L-lysine	Sigma	Cat#P1274
Laminin	Sigma	Cat#L2020
Leibovitz L-15 medium –Lys -Arg	GIBCO Life Technologies	N/A (customized)
Stable isotope-coded amino acids Lys4	Silantes GmbH	Cat#211103913
Stable isotope-coded amino acids Lys8	Silantes GmbH	Cat#211603902
Stable isotope-coded amino acids Arg6	Silantes GmbH	Cat#201203902
Stable isotope-coded amino acids Arg10	Silantes GmbH	Cat#201603902
Puromycin	Sigma	Cat#P8833
Sera-Mag Speed Beads A	GE Healthcare	Cat#24152105050250
Sera-Mag Speed Beads B	GE Healthcare	Cat#44152105050250
Trypsin/LysC	Promega	Cat#V5071

**Critical Commercial Assays**		

RNAqueous-Micro Total RNA Isolation Kit	Invitrogen	Cat#AM1931
OneStep RT-PCR kit	QIAGEN	Cat#210210
Duolink *in situ* PLA kit	Sigma	Cat#DUO92014

**Deposited Data**		

Proteomics data	This paper	PRIDE: PXD009250

**Experimental Models: Organisms/Strains**		

*X. laevis*	Nasco	https://www.enasco.com/p/9+-cm-Mature-Female-Xenopus-laevis-Frogs,-Live-Specimen+LM00535?fbclid=IwAR3sBev2jD7ZPRLqRfaecQb0P29WkPCI_Aov1xtHkNhmNbaIAHxRHGLnX9Y

**Oligonucleotides**		

Primer: β-actin	Sigma	N/A
*for* 5′ CCTGTGCAGGAAGATCACAT 3′		
*rev* 5′ TGTTAAAGAGAATGAGCCCC 3′		
Primer: Brn3a	Sigma	N/A
*for* 5′ TGAGCGATTCAAGCAGAGGAGG 3′	Sigma	N/A
*rev* 5′ TGCGACAGGGTGAGGGATTCAAAC 3′	Gene Tools	N/A
Primer: eIF2Bε	Gene Tools	N/A
*for* 5′ tgatgatgcaggcgctggaa 3′	Gene Tools	N/A
*rev 5′* caggtgaagcagggtggctttctg 3′		N/A
Morpholino: Control MO (CoMO)		N/A
5′ CCTCTTACCTCAGTTACAATTTATA 3′		N/A
Morpholino: PERK MO		N/A
5′ CGAACACTTTCACCTCATAACACTT 3′		N/A
Morpholino: Slit1 MO		N/A
5′ AGTAGTCTCAATGACACAATGACCA 3′		N/A

**Software and Algorithms**		

Volocity	v.6.3.1	RRID: SCR_002668
GraphPad PRISM	v.5.0c	RRID: SCR_002798
ImageJ	v.149	RRID: SCR_003070
DAVID	v.6.8	RRID: SCR_001881
KEGG	N/A	RRID: SCR_012773
String	v10.5	RRID: SCR_005223
Maxquant	v1.4.1.2	RRID: SCR_014485
Perseus	v1.5.1.6 or 1.5.5.3	RRID: SCR_015753

### Contact for Reagent and Resource Sharing

Further information and requests for resources and reagents should be directed to and will be fulfilled by the lead contact, Christine E. Holt (ceh33@cam.ac.uk).

### Experimental Model and Subject Details

#### *Xenopus laevis* embryos maintenance

*Xenopus laevis* embryos of either sex were obtained by *in vitro* fertilization as previously described (Campbell and Holt, 2001), raised in 0.1x modified Barth’s saline (MBS; 8.8 mM NaCl, 0.1 mM KCl, 0.24 mM NaHCO_3_, 0.1 mM HEPES, 82 μM MgSO_4_, 33 μM Ca(NO_3_)_2_, 41 μM CaCl_2_) at 14–22°C and staged according to Nieuwkoop and Faber (1994). This research has been regulated under the Animals (Scientific Procedures) Act 1986 Amendment Regulations 2012 following ethical review by the University of Cambridge Animal Welfare and Ethical Review Body (AWERB).

### Method Details

#### Retinal explant culture and axotomy assay on transwell filter

Whole eyes of anesthetized stage 35/36 embryos were dissected out and cultured at 20°C for 24h in 60% L15 minimal medium (Invitrogen), 1x Penicillin Streptomycin Fungizone on glass bottom dishes (MatTek) or on the top compartment of 6-well hanging inserts (Boyden chambers) with 1 μm membrane pores (Falcon), coated on both sides of the membrane with poly-L-lysine (10 μg/ml, Sigma) and only on the bottom side with laminin (10 μg/ml, Sigma).

For the pSILAC experiment 100 eye explants were cultured per condition, as detailed in Cagnetta et al. (2018). After 24 h, eye explants were pre-incubated with ISRIB for 30 min. Subsequently, eye explants were removed, scraped and washed off 7 times from the top compartment of the filter, leaving the somaless axons (∼2 μg protein typical yield) at the bottom. Sema3A or Sema3A and ISRIB were added, together with respective stable isotope-coded amino acids, to the somaless axons for 15 min. After stimulation the membrane was cut away, rinsed with ice cold PBS and lysed for protein extraction.

#### Pharmacological treatments

Stimulations were carried out using the following concentrations: Sema3A (150 ng/ml), Slit1 (200 ng/ml), ISRIB (200 nM), Thapsigargin (500 nM), Dithiothreitol (1 mM), GSK2606414 (300 nM), Cycloheximide (50 μM), PP242 (2.5 μM), U0126 (10 μM), Tautomycin (4nM).

#### Pulsed Stable Isotope Labeling by Amino acids in Cell culture

Experiments were performed in three independent biological replicates. Retinal explants were cultured in SILAC *light* medium (Lys0, Arg0) for 24 h and incubated in depletion medium (-Lys, -Arg) for 60 min prior pulse labeling. Subsequently, cell bodies were removed and somaless axons were incubated for 15 min with *medium* (M) (Lys4, Arg6) or *heavy* (H) isotope-coded amino acids (Lys8, Arg10). At 15 min samples were lysed, immediately pooled and processed by SP3.

#### Single-Pot Solid-Phase-enhanced Sample Preparation

Axons were harvested by the addition of lysis buffer (1% SDC, 0.1% SDS, 100mM TrisHCl ph 8.5, 10mM DTT, 1x protease inhibitor EDTA free). Samples were supplemented with 25 units Benzonase nuclease (Merck), and lysed in a Bioruptor (Diagenode) for 5 minutes (cycle 30/30, 4°C). Alkylation was performed by addition of 30 mM Chloroacetamide followed by incubation in the dark for 30 min. Protein clean-up, digestion and peptide clean-up were performed using a modified version of the ultrasensitive sample preparation protocol SP3 (Hughes et al., 2014). In brief, 5 μL of beads (1:1 mixture of hydrophilic and hydrophobic SeraMag Carboxylate-Modified beads, GE Life Sciences) were added to each sample. Acidified acetonitrile was added to achieve a final fraction of organic solvent of 50%. Beads were incubated for 10 min to allow complete binding of proteins to the beads. Protein clean-up was performed by subsequent wash with 70% Ethanol and once with Acetonitrile. For digestion, 0.1 μg sequencing grade Trypsin/LysC (Promega) was added and digestion was performed at 37°C for 16 h. Peptides were eluted with 9 μL 5% DMSO. 1 μL 10% formic acid was added and samples were stored at −20°C prior to MS analysis.

#### Mass Spectrometry

Samples were analyzed on a Orbitrap Velos Pro mass spectrometer (Thermo Scientific) using default settings. The mass spectrometer was coupled to a UPLC systems (Waters nanoAcquity UPLC). Peptides were loaded onto trap columns (Waters nanoAcquity Symmetry C_18_, 5 μm, 180 μm × 20 mm) with Buffer A (0.1% formic acid in water) and separated over a 25 cm analytical column (Acclaim PepMap RSLC, 75 μm × 2 μm) using 240 minute linear gradients from 3%–40% Buffer B (0.1% formic acid in Acetonitrile). MS2 Fragmentation was set to CID, and MSMS scans were acquired in the ion trap.

#### Proteomics data processing

Raw data were processed with Maxquant (version 1.4.1.2) (Cox and Mann, 2008) using default settings. MSMS spectra were searched against the *Xenopus laevis* Uniprot database (v20140925) concatenated to a database containing protein sequences of common contaminants. Raw data from Cagnetta et al. (2018) was used as a library to increase depth of identifications using the match-between-runs option, which was enabled in Maxquant. Enzyme specificity was set to trypsin/P, allowing a maximum of two missed cleavages. Cysteine carbamidomethylation was set as fixed modification, and methionine oxidation and protein N-terminal acetylation were used as variable modifications. The minimal peptide length was set to six amino acids. The mass tolerances were set to 20 ppm for the first search, and 4.5 ppm for the main search. Global false discovery rates for peptide and protein identification were set to 1%. The match-between-runs and re-quantify options were enabled.

#### Immunocytochemistry

Retinal cultures were fixed by paraformaldehyde except for anti-β-actin (AC-15 FITC) and anti-Gsn where methanol fixation was carried out. Secondary antibodies were species-specific dye-conjugated (Alexa Fluor, Invitrogen).

#### Puromycilation of NSPs

Retinal cultures were incubated with puromycin (2 ng/μl) for the condition and time (up to 15 min) of interest, fixed and incubated with anti-puromycin Alexa Fluor conjugate antibody.

Intact brains were incubated with puromycin (5 ng/μl) for 15 min in the condition of interest (Control or Tautomycin (20nM)), rinsed in culture medium, lysed and western blot anti-puromycin was carried out.

#### Puromycilation of NSPs and Proximity Ligation Assay

Retinal cultures were incubated with puromycin (2 ng/μl) for 10 min in the condition of interest, fixed and incubated with anti-puromycin and anti-Atf4 antibodies. Subsequently, Proximity Ligation Assay (PLA) was carried out using species-specific probes (tom Dieck et al., 2015).

#### Reverse Transcription Polymerase Chain Reaction

RNA was extracted from using RNAqueous-Micro Total RNA Isolation Kit. Primers were designed using *Primer3Plus* software. The annealing temperature used was 58°C for β-actin and Brn3a, 67°C for eIF2Bε.

#### Growth cone turning assay

Retinal explants from stage 35/36 embryos were cultured for 14-18 h on coverslips coated with poly-L-lysine (10 μg/ml) and laminin (10 μg/ml). Gradients of Sema3A (9 μg/ml) or control were generated by pulsatile ejection as described previously (Lohof et al., 1992, Campbell and Holt, 2001) for 60 min placing the micropipette at a starting distance equal to 100 μm and at an angle of 45**°** relative to the initial direction of the axon shaft. ISRIB (200 nM), GSK2606414 (300 nM), or Tautomycin (4nM) were bath-applied immediately prior to the start of the gradient assay.

For growth cone gradient assay the gradient was generated for 10 min placing the micropipette at 70 μm distance and at an angle of 90**°** relative to the growth cone and the initial direction of the axon shaft (Cagnetta et al., 2018). Subsequently samples were immediately fixed and immunostained for p-eIF2α.

#### Blastomere injection

*Xenopus* embryos were injected at the 4-cell stage in the dorsal animal blastomeres as previously described (Roque et al., 2016). 18 ng of PERK/Slit1/Control MO were injected into the blastomere of interest (Figure 6A).

#### DiI anterograde axon labeling

Stage 41 embryos were fixed in 4% formaldehyde in PBS at 4**°**C overnight. DiI solution was prepared by dissolving DiI powder (Thermo Scientific) in ethanol and injected into the eye cavity until completely filled. The embryos were incubated at room temperature for 48h to ensure complete dye diffusion. The brain was dissected and mounted in 1xPBS. The contralateral brain hemisphere was imaged.

#### Exposed brains

Stage 33/34 embryo brains were exposed by removing the overlying eye and epidermis (Wong et al., 2017) to ISRIB (2 μM) treatment at 22**°**C overnight, fixed and DiI injection was carried out.

#### Electroporation

Target eye electroporation was performed as previously described (Wong and Holt, 2018). The eye primordia of embryos stage 28 were injected with electroporation mixture (1 μg/μl pCS2+mGFP and 0.5 mM Control MO/PERK MO), followed by electric pulses of 50 ms duration at 1000 ms intervals, delivered at 18 V. The embryos were raised in 0.1x MBS until stage 45.

#### Western blot

Puromycilation assay of brains of stage 35/36 embryos and MOs specificity test on brain and eye tissue were carried out by western blot. Pierce BCA Protein Assay kit (Thermo Fisher Scientific) and spectrophotometry were used to determine the sample concentration. Bovine albumin serum (BSA, Invitrogen) was used to create a standard curve for protein concentration and for normalizing the concentration among samples. The antibody of interest was incubated at 4**°**C overnight in 5% BSA solution for the anti-Slit1 antibody, or 5% milk solution for the anti-puromycin and anti-PERK antibodies. The blots were then incubated with HRP-conjugated secondary antibodies (Abcam) at room temperature for 45 min, followed by ECL-based detection (Invitrogen).

### Quantification and Statistical Analysis

#### Statistics

Data were analyzed with PRISM 5 (GraphPad). Data are presented as mean and error bars represent s.e.m. Experiments were performed in at least three independent biological replicates. Details of statistical tests are presented in the figure legends.

^∗^p ≤ 0.05, ^∗∗^p ≤ 0.01, ^∗∗∗^p ≤ 0.001, ^∗∗∗∗^p ≤ 0.0001, ns: non-significant.

#### Bioinformatic data analysis

For protein quantification a minimum ratio count of 2 was set. The iBAQ was calculated to determine relative abundance levels of the pre-existing light-labeled proteins. Protein ratios were log2-transformed using the Perseus computational framework, and H/M ratios of NSPs were normalized to the median to center the distribution of ratios at 0 on the log2 scale, i.e., comparable numbers of proteins are upregulated and downregulated, respectively. To test whether the log2 ratio of each protein was significantly different from zero, p values were computed by a moderated t test implemented in the R/Bioconductor package limma (Ritchie et al., 2015). p values were corrected for multiple testing by controlling the false discovery rate with the method of Benjamini-Hochberg. Enrichment of categorical annotations (Gene Ontology) was determined using DAVID. Pathway and disease analyses were carried out using KEGG. Interaction network analysis was obtained by employing String v10.5 database. Each node represents a NSP change and each edge shows protein-protein interaction, disconnected nodes are not shown for simplicity. Upstream regulator analyses were carried out based on previous datasets identifying the targets of the following translational regulators: Apc, Mena, Fmrp, Tdp43, Fus, mTOR, PERK and GCN2 (Preitner et al., 2014, Vidaki et al., 2017, Darnell et al., 2011, Colombrita et al., 2012, Thoreen et al., 2012, Dang Do et al., 2009).

#### Quantification of Immunofluorescence

For the quantification of fluorescence intensity, isolated growth cones were selected randomly with phase optics. Low exposure was set up to avoid pixel saturation and the same gain and exposure settings were used for digital capture of images for each experiment which was performed in the same day, except for the growth cone gradient assay where the IF of the near and far sides within the same growth cone were compared. The outline of each single growth cone was traced using the phase image to define the region of interest (ROI) and the mean pixel intensity per unit area was measured in each channel using *Volocity* software. The background fluorescence was measured in a ROI as close as possible to the growth cone selected and subtracted to the mean fluorescence value of the growth cone. In the figures brightness/contrast settings were adjusted equally across images collected in the same experiment for presentation clarity.

For the growth cone gradient assay IF ratio analysis, the growth cone was bisected into two areas by a line drawn through the axon shaft and the background fluorescence level was subtracted. For the center of mass analysis, measurement was calculated as the average of all pixel locations weighted as intensity by using *ImageJ* software. The center of mass of the bright field was subtracted from the center of mass of the fluorescence signal.

#### Turning assay measurement

Turning angles were measured on growth cone images taken at 0 and 60 min using *ImageJ* software.

#### DiI quantification

For optic tract width quantification ten equally spaced concentric circles (C1-C10) were overlaid on the tract images with the center of the circles overlying the optic chiasm (OC) and C10 overlaying the Tectal Posterior Boundary (TPB) (Figure S6A). The widths of C2-4 and C5-8, corresponding respectively to pre- and post-caudal turn, were averaged. Lastly, the pre- and post-turn widths were normalized to the brain size, defined by the distance between OC and TPB. Mid-diencephalic turn (MDT) was measured as the angle between the pre-turn axon bundle (drawing a line from the optic chiasm and the ventral side of the MDT) and the post-turn axon bundle (drawing a line from the ventral side of the MDT and the tip of the most pioneer axon) (Figure 6B). The tectal projection angle (TPA) was measured as the angle between the post-turn tract and the most anteriorly projecting axon (Figure 6B). TPA was considered positive if pointing toward the posterior tectum, negative if pointing toward the anterior side of the tectum.

### Data and Software Availability

The accession number for the mass spectrometry proteomics data reported in this paper is ProteomeXchange Consortium via the PRIDE (Vizcaíno et al., 2016): PXD009250.

## Supplemental Information

Document S1. Figures S1–S6 and Table S2

Table S1. Axonal NSP Changes Induced by the Sema3A-p-eIF2α Pathway, Related to Figure 2, Figure S2, and Table S2

Document S2. Article plus Supplemental Information
