# Supplementary material for: Noncanonical Modulation of the eIF2 Pathway Controls an Increase in Local Translation during Neural Wiring
Source: Mol Cell. 2019 Feb 7;73(3):474–489.e5. doi: 10.1016/j.molcel.2018.11.013 (PMC6375727; doi:10.1016/j.molcel.2018.11.013)
Supplement: Document S1. Figures S1–S6 and Table S2 [file mmc1.pdf]

Molecular Cell, Volume *73*

Supplemental Information

Noncanonical Modulation of the eIF2 Pathway Controls an Increase in Local Translation during Neural Wiring

Roberta Cagnetta, Hovy Ho-Wai Wong, Christian K. Frese, Giovanna R. Mallucci, Jeroen Krijgsveld, and Christine E. Holt

Figure S1

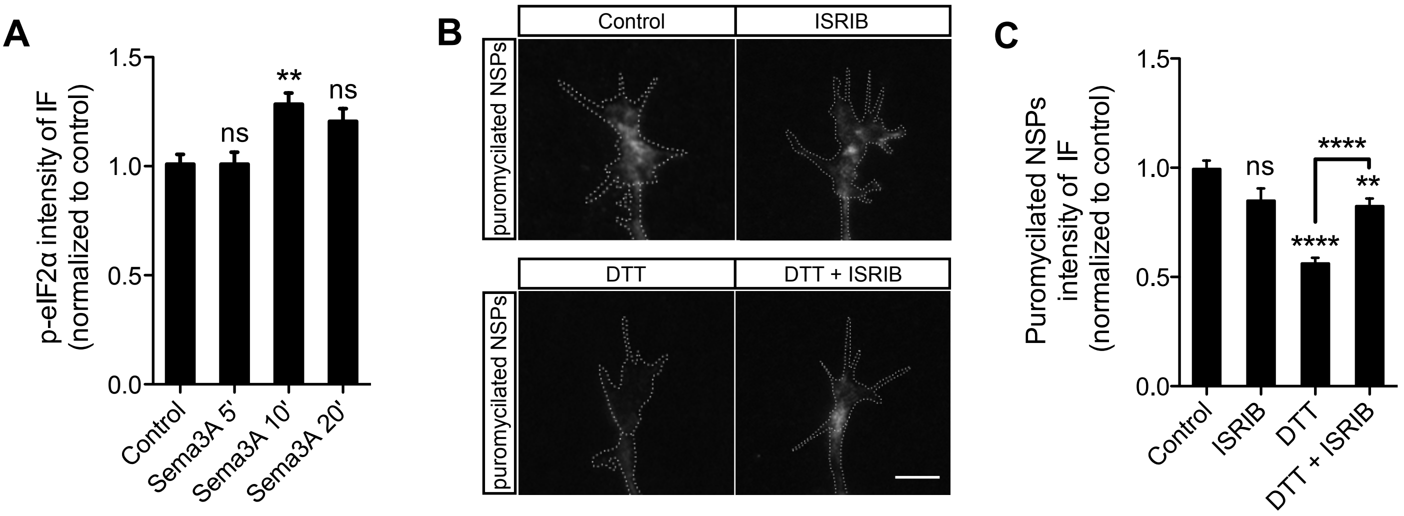

A
p-eIF2α intensity of IF
(normalized to control)
1.5
1.0
0.5
0.0
ns
**
ns
Control
Sema3A 5'
Sema3A 10'
Sema3A 20'
B
Control
ISRIB
puromycilated NSPs
DTT
DTT + ISRIB
puromycilated NSPs
C
Puromycilated NSPs
intensity of IF
(normalized to control)
1.5
1.0
0.5
0.0
ns
****
****
**
Control
ISRIB
DTT
DTT + ISRIB

Figure S1. Analysis of the DTT- and Sema3A-induced phosphorylation of eIF2α and its role in axonal translational control – Related to Figure 1 (A) Axons were treated with Sema3A for a timecourse stimulation (5 min, 10 min, 20 min), stained for p-eIF2α, and IF was measured (one-way ANOVA with Bonferroni's Multiple Comparison test). **(B-C)** Axons were incubated with puromycin and co-treated with DTT and ISRIB for 15 min, stained for puromycin, and IF was measured (one-way ANOVA with Bonferroni's Multiple Comparison test). For presentation clarity, brightness/contrast settings were adjusted equally across images collected in the same experiment. Error bars s.e.m. Scale bar 5 μm.

Figure S2

A

1 TTGGGAAGAA GGAAGGAGCG TAGGGTGTTA AGAGCTTAGT TTGTGGTTTT
51 TTTGTTAACG CGTATTAAAG GTTTGCGGAC GCCTATTGTA ACACGTTCCA
101 AGGATTTCTT CACTCCGGCG CCGCGATAAC AAGCGGAAAG CC**ATG**GCGCT
151 ATAGCGGCAG CAGAGGCCTG CGGCTCCCCT TACCACCGGC CAGCGCGAGG
201 CAGCAGCACC CAGTGGCGCT CGCAGCACCA GGCACAGCC**A** **TG**GCGCTTCT
251 CAAAGTAATC CACAGCAGCA TTGCTGTATA CGAGGAAGAA AGCTCAGAAT
301 CATCATCAGT CAAACTTCTG ATCCTACAGG AAA

B

1 TTTCTGCTTG CTGTCTGCCG GTTTAAGTTG TGTGCTCGGG TGTCCCTTTC
61 CTCTTCCCCT CCCGCAGGGC TTGCGGCCAC C**ATG**GCGTAT TAGAGGCAGC
101 AGTGCCTGCG GCAGCGTTGG CCTTTGCAGC GGCGGCAGCA GCACCAGGCT
151 CTGCAGCGGC AACCCCCACC GGCCTAAGCC **ATG**GCGCTCT TCACGAAATC
201 CAGCAGCAGT GTTGCTGTAA CGGACAAAGA TACCTTCGAG TTAAGCACAT
251 TCCTCGAATC CAGCAAAGCC CCACAAC

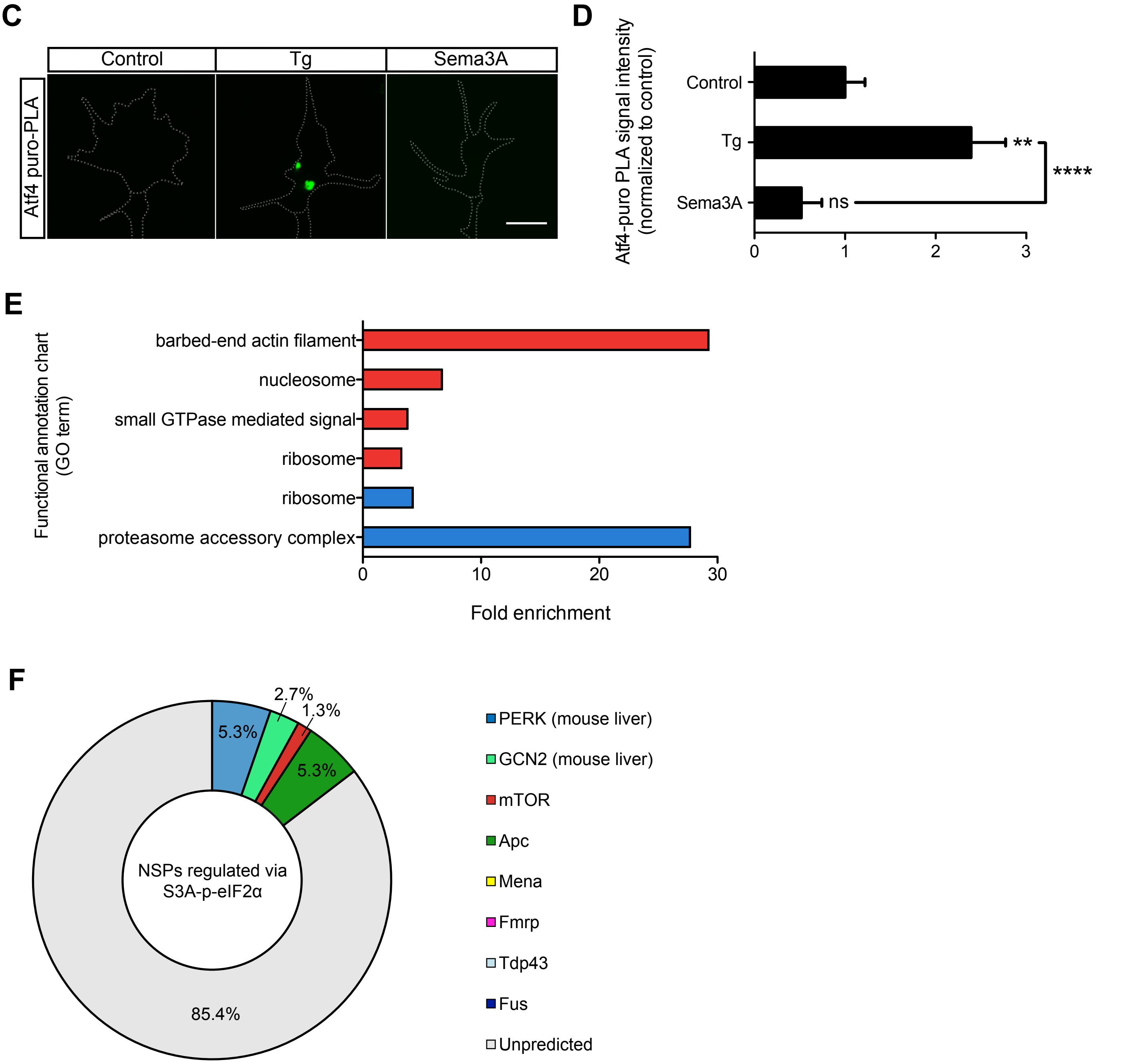

Figure S2. Sema3A-p-eIF2α signaling specifically remodels the nascent axonal proteome – Related to Figure 2 (A) The 5' UTR of Atf4 in *Xenopus laevis* conserves the two uORFs (indicated in bold) previously detected in mouse (Vattem and Wek, 2004). **(B)** The 5'UTR of Atf4 in mouse retinal axons (Shigeoka et al., 2016) exhibits the two uORFs previously detected in Mouse Embryo Fibroblast cells (Vattem and Wek, 2004). **(C-D)** Axons were treated with Tg or Sema3A for 1 h and incubated with puromycin over the last 10 min of the stimulation. PLA was carried out against puromycin and Atf4, and IF was measured (one-way ANOVA with Dunn's Multiple Comparison test). **(E)** Enriched Gene Ontology (GO) terms in the biological process, molecular function and cellular composition categories for the NSPs upregulated (indicated in red) and downregulated (indicated in blue) (p-value < 0.1). **(F)** Percentages of the Sema3A-p-eIF2α-induced NSP changes predicted to be targets of the *trans*-acting elements investigated (Thoreen et al., 2012; Preitner et al, 2014; Vidaki et al., 2017; Darnell et al, 2011; Colombrita et al., 2012; Dang do et al., 2009). For presentation clarity, brightness/contrast settings were adjusted equally across images collected in the same experiment. Error bars s.e.m. Scale bar 5 μm.

Figure S3

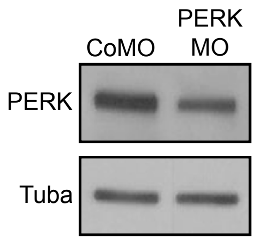

Figure S3. Validation of PERK MO – Related to Figure 3 Immunoblot of eye and brain lysates was probed for PERK in Control and PERK morphants.

Figure S4

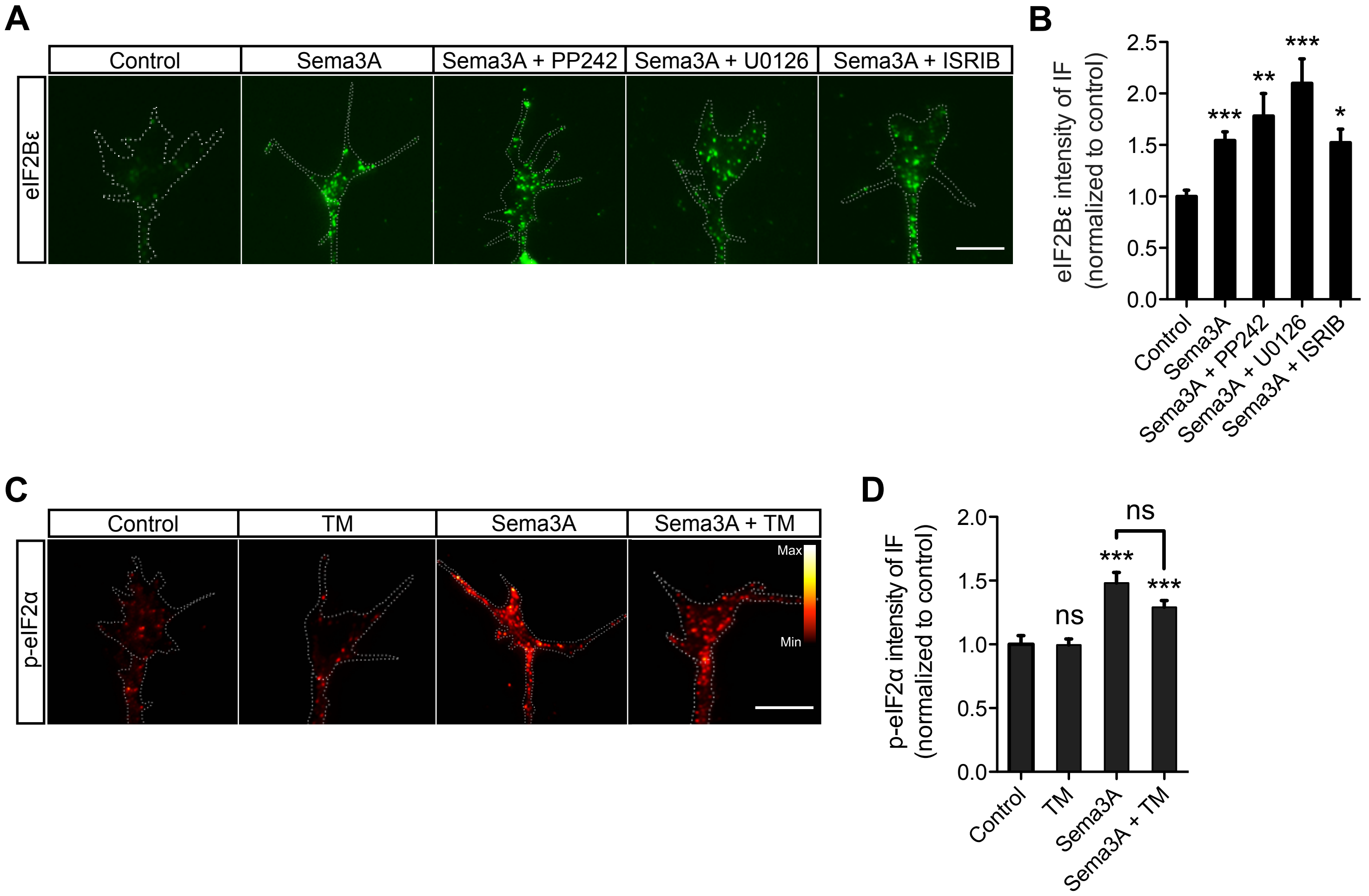

A
Control
Sema3A
Sema3A + PP242
Sema3A + U0126
Sema3A + ISRIB
eIF2Bε
B
eIF2Bε intensity of IF (normalized to control)
2.5
2.0
1.5
1.0
0.5
0.0
***
**
***
*
Control
Sema3A
Sema3A + PP242
Sema3A + U0126
Sema3A + ISRIB
C
Control
TM
Sema3A
Sema3A + TM
p-eIF2α
Max
Min
D
p-eIF2α intensity of IF (normalized to control)
2.0
1.5
1.0
0.5
0.0
ns
ns
***
***
Control
TM
Sema3A
Sema3A + TM

Figure S4. Investigation of Sema3A-eIF2Bε-p-eIF2α signaling – Related to Figure 4

(A-B) Axons were co-treated with Sema3A and PP242, U0126 or ISRIB for 5 min, immunostained for eIF2Bε, and IF was measured (one-way ANOVA with Dunn's Multiple Comparison test). **(C-D)** Axons were co-treated with Sema3A and TM for 10 min, stained for p-eIF2α, and IF was measured (one-way ANOVA with Dunn's Multiple Comparison test). For presentation clarity, brightness/contrast settings were adjusted equally across images collected in the same experiment. Error bars s.e.m. Scale bars 5 μm.

Figure S5

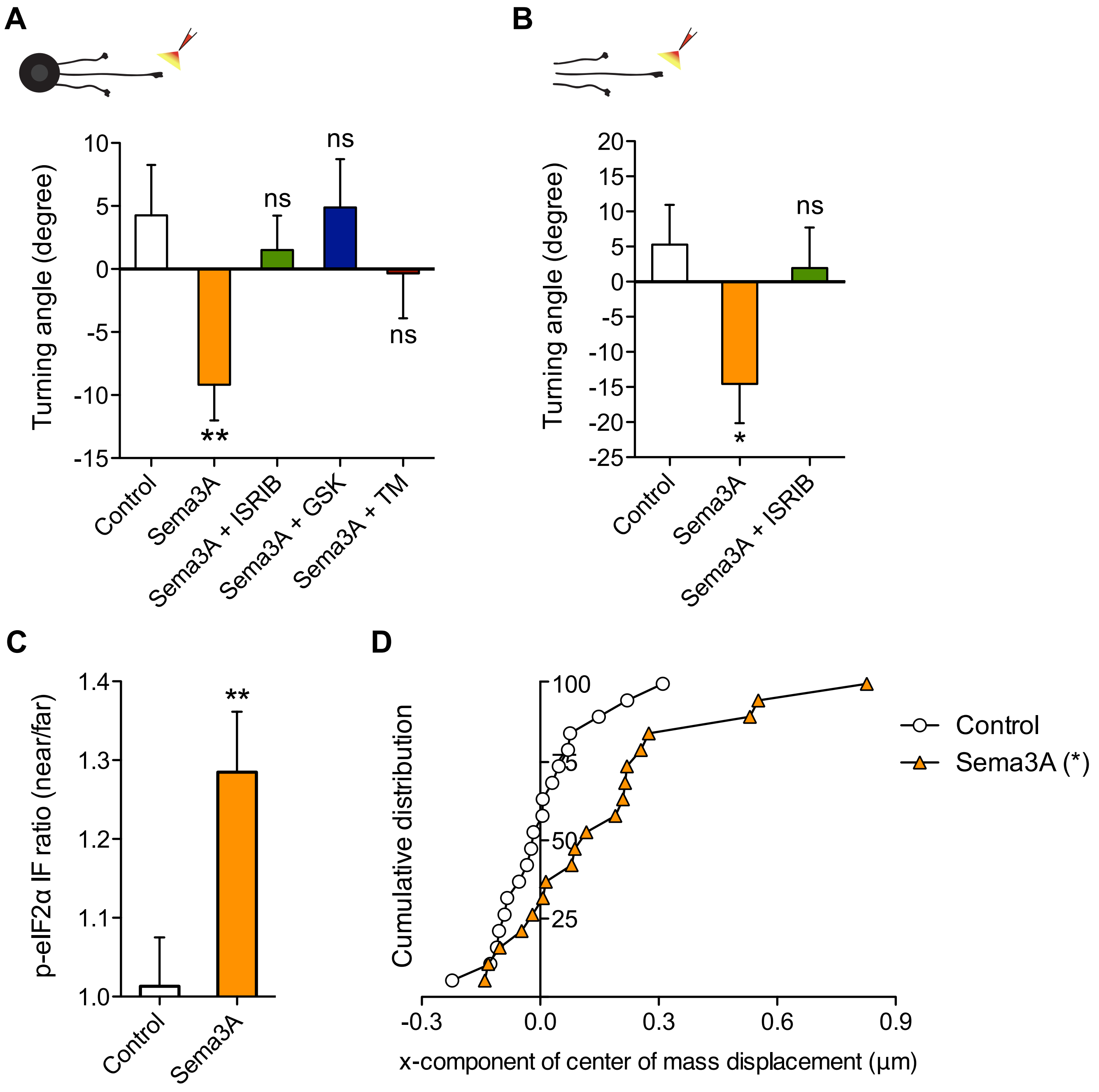

A
Turning angle (degree)
10
5
0
-5
-10
-15
ns
ns
ns
**
Control
Sema3A
Sema3A + ISRIB
Sema3A + GSK
Sema3A + TM
B
Turning angle (degree)
20
15
10
5
0
-5
-10
-15
-20
-25
ns
*
Control
Sema3A
Sema3A + ISRIB
C
p-eIF2α IF ratio (near/far)
1.4
1.3
1.2
1.1
1.0
**
Control
Sema3A
D
Cumulative distribution
100
75
50
25
-0.3
0.0
0.3
0.6
0.9
x-component of center of mass displacement (μm)
Control
Sema3A (*)

Figure S5. Spatially polarized phosphorylation of eIF2α mediates Sema3A-induced repulsive turning – Related to Figure 5 (A) Turning assay - a polarized gradient of Sema3A was generated and ISRIB, GSK or TM were bath-applied. Positive values indicate attraction, negative values indicate repulsion (Unpaired t-test). **(B)** Turning assay with somaless axons - a polarized gradient of Sema3A was generated and ISRIB was bath-applied. Positive values indicate attraction, negative values indicate repulsion (Unpaired t-test). **(C)** Asymmetric increase of p-eIF2α assessed by near/far ratio method (Unpaired t-test). **(D)** Cumulative distribution assessing asymmetric increase of p-eIF2α by center of mass method (Unpaired t-test). Error bars s.e.m.

Figure S6

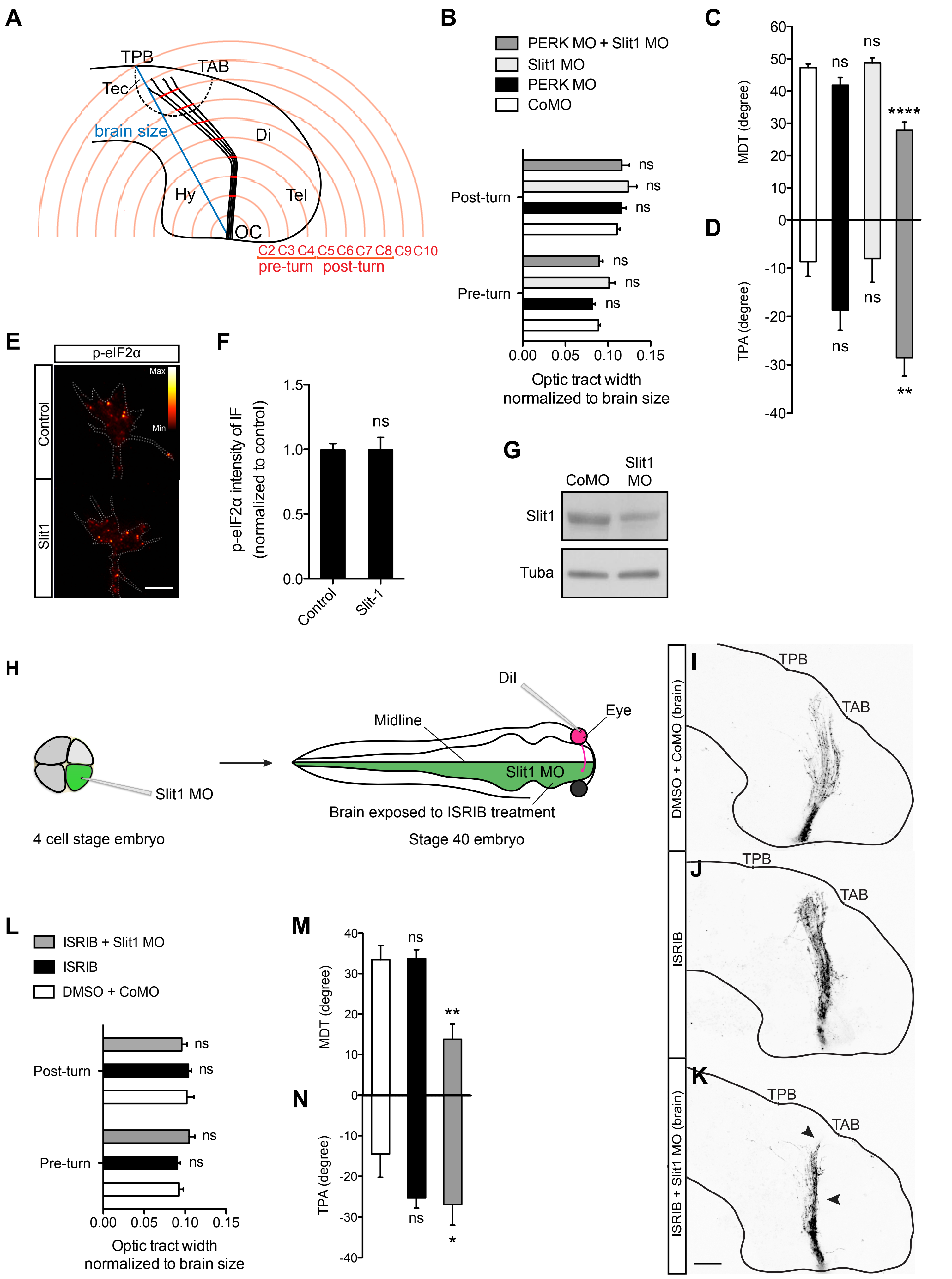

A
TPB
TAB
Tec
brain size
Di
Hy
Tel
OC
C2 C3 C4 C5 C6 C7 C8 C9 C10
pre-turn post-turn
B
PERK MO + Slit1 MO
Slit1 MO
PERK MO
CoMO
Post-turn
Pre-turn
ns
0.00 0.05 0.10 0.15
Optic tract width normalized to brain size
C
D
MDT (degree)
TPA (degree)
ns
****
**
E
p-eIF2α
Control
Slit1
Max
Min
F
p-eIF2α intensity of IF (normalized to control)
1.5
1.0
0.5
0.0
ns
Control
Slit-1
G
CoMO
Slit1 MO
Slit1
Tuba
H
Slit1 MO
4 cell stage embryo
DiI
Eye
Midline
Brain exposed to ISRIB treatment
Stage 40 embryo
I
DMSO + CoMO (brain)
J
ISRIB
K
ISRIB + Slit1 MO (brain)
L
ISRIB + Slit1 MO
ISRIB
DMSO + CoMO
Post-turn
Pre-turn
Optic tract width normalized to brain size
M
N
MDT (degree)
TPA (degree)
**
*

Figure S6. PERK-p-eIF2α signaling aids retinotectal axon navigation *in vivo* – Related to Figure 6 (A) Schematic illustrating the quantification of the width of the optic tract. Ten equally spaced concentric circles (C1-C10) were overlaid on the tract images with the center of the circles overlying the optic chiasm (OC) and C10 overlaying the Tectal Posterior Boundary (TPB). The widths of C2-4 and C5-8, corresponding respectively to pre- and post-caudal turn, were averaged. Lastly, the pre- and post-turn widths were normalized to the brain size, defined by the distance between OC and TPB. TAB, Tectal Anterior Boundary; Tec, tectum; Di, Diencephalon; Hy, Hypothalamus; Tel, Telencephalon. **(B)** Quantification of the pre- and post-turn width (two-way ANOVA). **(C)** MDT angle measurements (one-way ANOVA with Bonferroni's Multiple Comparison test). **(D)** TPA measurements - negative values indicate angles pointing towards the TAB (one-way ANOVA with Bonferroni's Multiple Comparison test). **(E-F)** Axons were treated with Slit1 for 10 min, stained for p-eIF2α, and IF was measured (Unpaired t-test). **(G)** Immunoblot of eye and brain lysates was probed for Slit1 in Control and Slit1 morphants. **(H)** Experimental outline to investigate the contribution of p-eIF2α and Slit1 to the axon navigation. Unilateral Slit1 MO injection leaded to a targeted KD in half of the CNS, which was subsequently exposed to ISRIB treatment. **(I-K)** DiI-filled stage 40 retinotectal projections. **(L)** Quantification of the pre- and post-turn width (two-way ANOVA). **(M)** MDT angle measurements (one-way ANOVA with Dunn's Multiple Comparison test). **(N)** TPA measurements – positive values indicate angles pointing towards the TPB, negative values indicate angles pointing towards the TAB (one-way ANOVA with Dunn's Multiple Comparison test). For presentation clarity, brightness/contrast settings were adjusted equally across images collected in the same experiment. Error bars s.e.m. Scale bars 5 μm (E) and 100 μm (I-K).

Table S1. Axonal nascent proteins regulated by Sema3A-p-eIF2α signaling – Related to Figure 2

Table S2

**Neurological Disease**	**NSPs regulated by Sema3A-p-eIF2α signaling**
Amyotrophic lateral sclerosis	Sod1, Vcp, Hnrnpa1
Hereditary spastic paraplegia	L1cam, Hspd1
Charcot-Marie-Tooth disease	Vcp
Hypomyelinating leukodystrophy	Hspd1
Syndromic X-linked mental retardation	L1cam
Frontotemporal lobar degeneration	Vcp
Cerebral dysgenesis, neuropathy, ichthyosis, and palmoplantar keratoderma syndrome	Snap29
Early infantile epileptic encephalopathy	Mdh2
ATP synthase deficiency	Atp5a1
Congenital hydrocephalus	L1cam
Hereditary sensory and autonomic neuropathy	Cct5
Familial amyloidosis	Gsn
L1 syndrome	L1cam
Juvenile-onset dystonia	Actb

Table S2. Axonal NSP changes induced by Sema3A-p-eIF2α signaling associated with neurological disorders – Related to Figure 2 Neurological disorders were selected from the *KEGG disease* output.
